# Poplar carbohydrate‐active enzymes: whole‐genome annotation and functional analyses based on RNA expression data

**DOI:** 10.1111/tpj.14417

**Published:** 2019-07-01

**Authors:** Vikash Kumar, Matthieu Hainaut, Nicolas Delhomme, Chanaka Mannapperuma, Peter Immerzeel, Nathaniel R. Street, Bernard Henrissat, Ewa J. Mellerowicz

**Affiliations:** ^1^ Umeå Plant Science Center Department of Forest Genetics and Plant Physiology Swedish University of Agricultural Sciences Umea Sweden; ^2^ Architecture et Fonction des Macromolécules Biologiques Centre National de la Recherche Scientifique (CNRS) Aix‐Marseille University Marseille France; ^3^ INRA USC 1408 AFMB Marseille France; ^4^ Umeå Plant Science Center Plant Physiology Department Umeå University Umeå Sweden; ^5^ Chemical Engineering Karlstad University Karlstad 65188 Sweden

**Keywords:** carbohydrate metabolism, cell wall, comparative genomics, genome sequencing, vegetative development, wood formation

## Abstract

Carbohydrate‐active enzymes (CAZymes) catalyze the formation and modification of glycoproteins, glycolipids, starch, secondary metabolites and cell wall biopolymers. They are key enzymes for the biosynthesis of food and renewable biomass. Woody biomass is particularly important for long‐term carbon storage and as an abundant renewable natural resource for many industrial applications. This study presents a re‐annotation of CAZyme genes in the current *Populus trichocarpa* genome assembly and *in silico* functional characterization, based on high‐resolution RNA‐Seq data sets. Altogether, 1914 CAZyme and expansin genes were annotated in 101 families. About 1797 of these genes were found expressed in at least one *Populus* organ. We identified genes involved in the biosynthesis of different cell wall polymers and their paralogs. Whereas similar families exist in poplar and *Arabidopsis thaliana* (with the exception of CBM13 found only in poplar), a few families had significantly different copy numbers between the two species. To identify the transcriptional coordination and functional relatedness within the CAZymes and other proteins, we performed co‐expression network analysis of CAZymes in wood‐forming tissues using the AspWood database (http://aspwood.popgenie.org/aspwood-v3.0/) for *Populus tremula*. This provided an overview of the transcriptional changes in CAZymes during the transition from primary to secondary wall formation, and the clustering of transcripts into potential regulons. Candidate enzymes involved in the biosynthesis of polysaccharides were identified along with many tissue‐specific uncharacterized genes and transcription factors. These collections offer a rich source of targets for the modification of secondary cell wall biosynthesis and other developmental processes in woody plants.

## Significance Statement

Re‐annotation of carbohydrate‐active enzymes in poplar and their transcript analyses reveal genes and gene families involved in wood cell wall biosynthesis.

## Introduction

Carbohydrate‐active enzymes (CAZymes) are essential proteins for all life forms (Lombard *et al*., 2014). They are fundamentally important for the synthesis, degradation and modification of all the glycoconjugates found in nature, including carbohydrates, glycoproteins, glycolipids and glycosylated secondary metabolites (Wilson, 2002; Cantarel *et al*., 2009). Plant tissues are particularly rich in complex carbohydrates, building the cell walls and thus affecting growth and development. Therefore, plants depend heavily on carbohydrate metabolism for a diverse array of physiological processes, including growth, defense, dormancy, signaling and photosynthesis (Coutinho *et al*., 2003). As a result of high abundance and sustainable carbon assimilation from the atmosphere, the carbohydrate‐rich tissues of plants have become important feedstocks for biofuel and biomaterial production (Himmel *et al*., 2007).

The classification of CAZymes into families is a difficult task, as different parameters need to be considered simultaneously, including a significant similarity of amino acid sequences with at least one biochemically characterized family member, the presence of defined catalytic and non‐catalytic modules, and the availability of a full‐length protein sequence (Lombard *et al*., 2014). By adopting this approach, the CAZymes were previously classified into four major classes, namely glycosyltransferases (GTs), glycoside hydrolases (GHs), polysaccharide lyases (PLs) and carbohydrate esterases (CEs) (Cantarel *et al*., 2009). Recently, the lignin degrading enzymes and the polysaccharide lytic monooxygenases have been added and classified as auxiliary activity (AA) families, which encompass a broad range of enzymes related to lignocellulose conversion (Levasseur *et al*., 2013). In plants, this group also contains diverse enzymes involved in biosynthesis and developmental processes. In addition, the carbohydrate binding modules (CBMs) encompass a group of proteins with specific domains that bind to carbohydrates (Boraston *et al*., 2004), for example to substrates, as in the case of hydrolytic enzymes (Guillen *et al*., 2010), or to ligands, as in in case of receptors (Galindo‐Trigo *et al*., 2016). These six categories in total are organized in sequence‐based families in the CAZy database (http://www.cazy.org).

As primary producers of terrestrial ecosystems, woody plants play a special role in providing an ecologically essential carbon sink, as well as serving as an important source of industrial raw material. A major proportion of assimilated carbon in woody species is used for cambial growth and the development of the secondary cell walls in xylem cells. Once the carbon is allocated to cellulose, lignin or, to a large extent, hemicelluloses in xylem cell walls, it is immobilized for many decades or even centuries. Therefore, the carbohydrate‐rich secondary cell walls of woody biomass are particularly important as a renewable natural resource for CO_2_‐neutral industrial applications, with the goal of replacing fossil‐based resources (Grattapaglia *et al*., 2009; Hinchee *et al*., 2009). As CAZymes are involved in the formation and modification of this carbohydrate matrix (Mellerowicz and Sundberg, 2008), a detailed study of CAZymes in a woody model species and their spatial interactions during wood cell wall formation is essential to inform tree improvement programs to support the future bioeconomy.

The availability of a mature genome assembly (v3.0) for *Populus trichocarpa*, together with recent high‐resolution RNA‐Seq data sets for *Populus* sp. (Sundell *et al*., 2015, 2017; Immanen *et al*., 2016), prompted us to carry out a re‐annotation and expression analysis of CAZymes in *Populus*. Here, we redefined all members of different CAZyme families and closely related expansins, and compared their genetic diversity with our earlier *P. trichocarpa* v1.0 annotation (Geisler‐Lee *et al*., 2006) and with the current *Arabidopsis thaliana* (http://www.cazy.org) annotation. Furthermore, we have annotated CAZyme families in *P. trichocarpa* and their orthologs in *A. thaliana*. The available *Populus* RNA‐Seq data sets were used to determine tissue specificity, and the co‐expression network analyses among the CAZymes and other genes enabled the discovery of additional gene models with putative function in wood cell wall biosynthesis.

## Results and discussion

### Identification of CAZyme families

The comparison of protein‐coding transcripts of *P. trichocarpa* gene models (v3.0) with CAZyme and expansin modules in the CAZy database resulted in the identification of 1914 CAZyme and expansin gene models, including 629 GHs, 788 GTs, 42 PLs, 106 CEs and 65 expansin‐related sequences. Two other CAZyme superfamilies, the recently added catalytically active AAs (112) and the non‐catalytic CBMs (173, excluding those appended to GH and GT families), were also identified. All of the genes identified are listed in Tables S1–S7.

Compared with the previous annotation (Geisler‐Lee *et al*., 2006), the current annotation includes more families in GTs, GHs and CEs. Thus, we have identified 42 out of 106 GT families, 37 out of 156 GH families, two out of 29 PL families, four out of 16 CE families, four out of 15 AA families and 12 out of 84 known CBM families listed in the CAZy database (as found in November 2018) (Table 1). *Populus* has all the CAZyme families identified in *A. thaliana*, whereas one family found in *Populus*, CBM13, does not have counterparts in *A. thaliana* (Table 1). This domain has a trefoil structure, with three sugar binding domains that can harbor different specificities to mono‐ or disaccharides and that belong to the ricin B lectin family (Fujimoto, 2013). All identified *P. trichocarpa* CBM13 gene models clustered at two loci on chromosome 6, and were similar to ricin and abrin‐a, potent plant toxins.

**Table 1:** CAZyme families and the number of CAZymes models per family detected in *Populus trichocarpa* v3.0 and *Arabidopsis thaliana* v10.0 genomes (November 2018)

CAZyme families	*Ath*	*P. trichocarpa*		*Ath* vs *Pt* *Q* values	CAZyme families	*Ath*	*P. trichocarpa*		*Ath* vs *Pt* *Q* values
	v.10	v.3	v.3 vs v.1			v.10	v.3	v.3 vs v.1	
AA1	36^a^	78^a^	78	3.3*	*(Continued)*				
AA5	7^a^	11^a^	11	0.1	GH63	2	1	−2	0.9
AA6	4^a^	9^a^	9	0.5	GH77	2	3	0	0.1
AA7	27^a^	14^a^	14	11.6**	GH79	3	6	−1	0.2
CE6	2	4^a^	4	0.2	GH81	2	1	−3	0.9
CE8	67	89	0	0.7	GH85	2	2^a^	2	0.2
CE11	4^a^	2^a^	2	1.8	GH89	1	3	1	0.4
CE13	12	11	1	1.5	GH95	1	4	0	0.9
PL1	26	31	3	0.8	GH100	9	16	−3	0.2
PL4	8	11	0	0.1	GH116	4	5^a^	5	0.1
EXPN	36	65	23	0.8	GH146	2	3^a^	3	0.1
(CBM13)_1–2_	0	6	6	4.0**	GH152	19	40^a^	40	1.5
CBM18‐(EXPN)_0–1_	1	5	5	0.9	GT1	122	281	−45	15.6**
CBM18‐GH19	9	15^a^	15	0.1	GT2	42	60	−14	0.1
CBM20	2	7^a^	7	1.2	GT4	24	38	−3	0.1
(CBM20)_2_‐GH77	1	2^a^	2	0.1	GT5	6	7	−6	0.3
(CBM22)_1–4_‐GH10	11	8^a^	8	2.6	GT8	42	56	−5	0.4
CBM32	1^a^	1^a^	1	0.1	GT10	3	4	0	0.1
CBM43	31	34^a^	34	1.7	GT13	1	2	−1	0.1
GH17‐CBM43	28	43^a^	43	0.1	GT14	11	16	2	0.1
CBM45	2	2^a^	2	0.2	GT16	1	1	0	0.1
(CBM45)_2_‐GH13	1	1^a^	1	1.6	GT17	7	5	0	1.8
CBM48	8	18^a^	18	0.9	GT19	1	1	0	0.1
(CBM48)_1–2_‐GH13	7	10	10	0.1	GT20	11	13	0	0.4
GH9‐CBM49	3	3^a^	3	0.3	GT22	3	5	1	0.1
CBM50	1	12^a^	12	5.7**	GT24	1	1	−1	0.1
(CBM53)_3_‐GT5	3	1^a^	1	2.1	GT28	4	5	−1	0.1
CBM57	4	92^a^	92	51.1**	GT29	3	5	0	0.1
GH1	48	49	−1	3.8*	GT30	1	1	0	0.1
GH2	2	3	0	0.1	GT31	33	48	−4	0.1
GH3	16	27	−6	0.2	GT32	6	5	−1	1
GH5	13	24	2	0.4	GT33	1	1	0	0.1
GH9	29	32	−1	1.6	GT34	8	9	1	0.4
GH10	12	8	1	3.4*	GT35	2	5	−1	0.4
GH13	10	16	−5	0.1	GT37	10	7	−1	2.6
GH14	9	14	2	0.1	GT41	2	4	−1	0.2
GH16	33	43	2	0.5	GT43	4	7	0	0.1
GH17	51	88	−9	0.6	GT47	39	70	−5	0.8
GH18	11	28	−2	2.3	GT48	13	13	−6	1.2
GH19	14	21	0	0.1	GT50	1	1	0	0.1
GH20	3	6	0	0.2	GT57	2	3	−1	0.1
GH27	4	9	1	0.5	GT58	1	1	0	0.1
GH28	68	85	−4	1.4	GT59	1	1	0	0.1
GH29	1	3	0	0.4	GT61	8	10	0	0.2
GH31	5	12	−2	0.8	GT64	3	5	1	0.1
GH32	8	8	−1	0.7	GT66	2	4	−2	0.2
GH33	1	1^a^	1	0.1	GT75	5	10	−1	0.3
GH35	18	23	1	0.3	GT76	1	2^a^	2	0.1
GH36	6	17	−2	1.9	GT77	19	19	3	1.7
GH37	1	4	0	0.9	GT90	9	7^a^	7	1.8
GH38	4	9	1	0.5	GT92	5	3^a^	3	1.8
GH43	2	1	−1	0.9	GT95	3	6^a^	6	0.2
GH47	5	6	‐1	0.2	GT96	2	1^a^	1	0.9
GH51	2	6	3	0.8	GT106	34	44^a^	44	0.5

a Families in *P. trichocarpa* not reported by Geisler‐Lee *et al*. (2006) or in *A. thaliana* not listed in the CAZy database (http://www.cazy.org/). The identified *A. thaliana* genes include CBM32 (At5g49570) and AA family genes listed in Table S8.

* and **, families differing in relative gene frequencies between *P. trichocarpa* and *A. thaliana*, taking into account the total number of genes in these species, 41377 and 27416, respectively (*P ≤ 0.10; **P ≤ 0.05, χ^2^ test).

### Major changes of CAZyme gene families in *P. trichocarpa* v3.0 versus v1.0 assembly

The current *P. trichocarpa* v3.0 genome contains more CAZymes and expansin gene models (1914) than were reported for the previous v1.0 assembly (1603; Geisler‐Lee *et al*., 2006). This is essentially linked to the expansion of the CAZyme database, in which several families have been added or expanded since the first analysis. Considering the previously annotated families (Geisler‐Lee *et al*., 2006), the total number of genes has been reduced by 7% on average. For example, GT2 lost 14 members, whereas GT8 and GT47 each lost five gene models. This reduction of family members is the result of the significantly improved method of coding sequence detection and improved genome assembly. The lists of v1.0 gene models annotated as CAZymes coinciding (at least partially) with the current CAZyme models are included in Tables S1–S4 and S6, and the change in the number of genes in each family is listed in Table 1.

### Genetic diversity of CAZyme gene families in *Populus* compared with *A. thaliana*

Approximately 1.6 times more CAZyme genes were identified in *P. trichocarpa* (v3.0) than in *A. thaliana*, as listed in the CAZy database in November 2018 (http://www.cazy.org) and supplemented by our homology‐based annotation of *A. thaliana* AAs (Table S8). This increase was similar to that previously reported (Geisler‐Lee *et al*., 2006). This observation is in line with the census of CAZyme motifs carried out in the genomes of several streptophytes, including green algae, a moss and many vascular plants, and shows that whereas the woody plants appear to have the highest absolute numbers of CAZyme motifs in their genomes, the frequencies of these domains in relation to respective genome size are stable (Pinard *et al*., 2015). Thus, the difference in the absolute number of CAZymes between *Populus* and *A. thaliana* is related to the linage‐specific history of whole‐genome duplication and gene loss (Tuskan *et al*., 2006).

Nevertheless, the comparative analysis of relative gene abundance (in relation to genome size) in each CAZyme family between *A. thaliana* and *P.  trichocarpa* showed significant differences in a few families (Table 1). For example, the GT1 family, responsible for the glycosylation of different metabolites, the AA1 family, comprising lignification‐related laccases, the CBM50 family, known also as LysM domain binding chitin, and the CBM57 family, including various leucine‐rich receptor‐like kinases (LRKs) were over‐represented in *P. trichocarpa*. Conversely, the GH1 family of diverse exoglycanases, the GH10 family containing xylanases, the GH17 family involved in callose metabolism and the AA7 family comprising the berberine bridge enzymes were proportionally over‐represented in *A. thaliana*. These differences provide data for hypotheses on adaptations to perennial/woody versus annual/herbaceous lifestyles, which can be tested by comparative genome analyses.

### CAZyme families identified in *Populus* since the last whole‐genome annotation

#### Glycosyltransferases

The glycosyltransferases (GTs; EC 2.4.X.X) play a major role in the biosynthesis of cell wall polymers and starch, and the glycosylation of proteins, lipids and secondary metabolites. We annotated six GT families in the *P. trichocarpa* v3.0 genome assembly that were not annotated in v1.0 assembly (Geisler‐Lee *et al*., 2006) (Table 1). The GT76 family includes fungal and human mannosyl transferases that use dolichol‐*P*‐mannose as a donor to synthesize the glycophosphatidylinositol (GPI) anchor (Kang *et al*., 2005). The GPI anchor proteins are abundant in plants, and GT76 might have a similar role in this kingdom. The GT90 family includes β‐1,2‐xylosyltransferases involved in protein glycosylation and in the biosynthesis of glucuronoxylomannan and galactoxylomannan found in fungal capsules (Klutts *et al*., 2007).

The remaining four GT families, GT92, GT95, GT96 and GT106, have been created since the previous *Populus* CAZyme analysis and they represent different activities based on studies in *A. thaliana*. GT92 members are UDP‐galactose 4‐galactosyltransferases that synthesize rhamnogalacturonan‐I (RG‐I) β‐1,4‐d‐galactan (Liwanag *et al*., 2012). GT95 members have been characterized as hydroxyproline *O*‐arabinosyl transferases (HPATs) catalyzing the transfer of l‐arabinofuranosyl (Ara*f*) residue from UDP‐β‐L‐Ara*f* to the hydroxyl group of the hydroxyproline residues of extensins (Ogawa‐Ohnishi *et al*., 2013), peptide hormones (Schnabel *et al*., 2011; Okamoto *et al*., 2013; Xu *et al*., 2015) and possibly other proteins. The GT96 family comprises serine *O*‐α‐galactosyltransferases (SGTs) (Saito *et al*., 2014) involved in the post‐translational modification of arabinogalactan proteins (AGPs) and extensins. It is represented by a single model in *P. trichocarpa*, and the protein was previously annotated in the CAZy database (http://www.cazy.org). GT106 is a recently created CAZyme family, one clade of which contains RG‐I:rhamnosyltransferases (RRTs) involved in the biosynthesis of the RG‐I backbone (‐4GalUAα1‐2Rhaα1‐)_n_ (Takenaka *et al*., 2018). Three out of 44 GT106 *Populus* sequences had previously been deposited in the CAZy database (http://www.cazy.org).

#### Glycoside hydrolases

Glycoside hydrolases (EC 3.2.1.X) catalyze the hydrolysis of *O*‐ or *S*‐glycosidic bonds and have a major role in cell wall remodeling and architecture, carbohydrate metabolism and protein post‐translational modifications, affecting the physiological activities of plant cells. Five additional GH families were annotated since the previous analysis (Geisler‐Lee *et al*., 2006) (Table 1). One of these, GH85, has been characterized in *A. thaliana* as cytosolic endo‐*N*‐glucosaminidase that cleaves *O*‐glycosidic linkage between two *N*‐acetylglucosamines of *N*‐glycans (Fischl *et al*., 2011). Four other families have been studied in organisms other than plants. GH33 comprises sialidases (EC 3.2.1.18) cleaving α‐ketosidic linkage between the sialic (*N*‐acetylneuraminic) acid and a sugar residue in prokaryotes, fungi and animals. Sialic acid is not found in plants, but its derivatives, KDO and 2‐keto‐3‐deoxy‐d‐lyxo‐heptulosaric acid (DHA) are present in the side chain C and D of rhamnogalacturonan‐II (RG‐II), and could perhaps serve as substrates for plant GH33 members. The GH116 family includes diverse activities such as β‐glucosidase (EC 3.2.1.21), β‐xylosidase (EC 3.2.1.37), acid β‐glucosidase/β‐glucosylceramidase (EC 3.2.1.45) and β‐*N*‐acetylglucosaminidase (EC 3.2.1.52), and is found in all life domains. GH146 and GH152 are recently created GH families in the CAZy database. The GH146 family comprises β‐l‐arabinofuranosidase (EC 3.2.1.185) characterized in bacteria of human gut cleaving both β‐1,2‐ and β‐1,3‐linked l‐Ara*f* branches recently identified in pectic arabinan (Luis *et al*., 2018). The GH152 family groups stress‐induced thaumatin‐like proteins that are highly abundant in plants (Trudel *et al*., 1998). Seven out of 40 *Populus* GH152 members have already been annotated in the CAZy database (http://www.cazy.org). A fungal GH152 member has been shown to have β‐1,3‐glucanase (EC 3.2.1.39) activity (Sakamoto *et al*., 2006). Plant GH152 proteins exhibit callose‐binding activity (Trudel *et al*., 1998), suggesting that they could also be β‐1,3‐glucanases.

#### Carbohydrate esterases

The carbohydrate esterases (CEs) are involved in the remodeling and degradation of the plant cell wall polysaccharides. Plant genomes are known to contain pectin methyl esterases (PMEs, EC 3.1.1.11) in family CE8, and pectin acetyl esterases (EC 3.1.1.X) in family CE13, with both families being previously annotated in *P. trichocarpa* (Geisler‐Lee *et al*., 2006). Since then, one member of family CE6 has been annotated in *Populus* (http://www.cazy.org), whereas we report here three additional CE6 members (Table 1). CE6 contains microbial xylan acetyl esterases (Neumüller *et al*., 2015), but its function in plants has not been demonstrated. CE11 members are annotated in *A. thaliana* as UDP‐3‐*O*‐acyl *N*‐acetylglucosamine deacetylases that are likely to be involved in lipid biosynthesis via a recently discovered pathway resembling that of lipid A biosynthesis in bacteria (Li *et al*., 2011a). We have identified two members of CE11 in *P. trichocarpa* (Table 1). The CAZy database additionally lists one CE4 member annotated as *Populus trichocarpa *×* deltoides* ABK96767. This sequence is not homologous to any gene model in *P. trichocarpa* v3.0, but is instead highly similar to a rust *Melampsora larici‐populina* 98AG31 CE4 protein XP_007418383. Thus, it appears that the CE4 family is not present in *Populus*.

#### Subfamilies of polysaccharide lyases

Polysaccharide lyases (PLs) (EC 4.2.2.‐) cleave 4‐*O*‐glycosidic bonds in pectins and glucosaminoglycans using a β‐elimination mechanism without using a water molecule (Yip and Withers, 2006). They are involved in diverse biochemical processes, including biomass degradation, cell wall matrix recycling and pathogenesis, and are largely substrate specific (Herron *et al*., 2000; Abbott and Boraston, 2008). Plants have members in PL1, characterized as pectate lyases (PELs; EC 4.2.2.2) (Domingo *et al*., 1998; Wang *et al*., 2010; Biswal *et al*., 2014), and in PL4, annotated as rhamnogalacturonan lyases (EC 4.2.2.23), that affect cell separation and pectins in middle lamella (Molina‐Hidalgo *et al*., 2013), but the actual activity has not been demonstrated for any PL4 plant protein. The lack of critical catalytic residues in many of the plant PL4 proteins has led to doubts regarding their enzymatic activity (Kozlova *et al*., 2017). Based on sequence similarity and substrate specificity, the PL families were further divided into subfamilies represented by an Arabic numeral following the family identifier (Lombard *et al*., 2010). The current *Populus* CAZyme annotation identified PL1_1, PL1_12, and PL4_2 subfamilies (Table S3). PL1_1 and PL1_12 both include pectate lyases but PL1_12, classified as clade V of the pectate lyase family, does not have a Ca^2+^ binding site or other motifs present in PL1_1 (Bai *et al*., 2017).

#### Auxiliary activities

The category broadly termed as ‘auxiliary activities’ (AAs) has been introduced to the CAZy database since the previous *Populus* genome‐wide annotation (Geisler‐Lee *et al*., 2006). It groups together the families of redox enzymes and lytic polysaccharide monooxygenases (LPMOs) involved in polysaccharide, oligosaccharide and lignin breakdown, lignin polymerization and other metabolic processes (Levasseur *et al*., 2013). The *P. trichocarpa* v3.0 genome assembly contains four AA families (Tables 1 and S5), similar to *A. thaliana*. Not all *A. thaliana* members have been annotated in the current CAZy database, however. Therefore, *P. trichocarpa* sequences were used to identify the corresponding proteins in *A. thaliana* by amino acid sequence alignment and phylogenetic analysis (Figure S1; Table S8).

The AA1 family, annotated as multicopper oxidase, is the most abundant *Populus* AA family (Figure S1; Table 1). It includes a large subfamily of laccases that are considered as key lignin polymerizing enzymes, possessing *p*‐diphenol:O_2_ oxidoreductase activity (EC 1.10.3.2), although only a few members have been shown to carry out this function (Turlapati *et al*., 2011; Lu *et al*., 2013), and a large subfamily of *SKU5‐SIMILAR* (*SKS*) proteins with GPI anchor that are implicated in stress responses and the regulation of development (Sedbrook *et al*., 2002). Forty‐nine laccases were annotated in *P. trichocarpa* (Lu *et al*., 2013) and our analysis identified 10 additional gene models, some of which might be truncated (Table S5). The AA1 family is abundantly represented in the CAZy database, with 56 entries for *P. trichocarpa* and several for *Populus alba*,* Populus fremontii*,* Populus nigra* and *Populus tomentosa* (http://www.cazy.org). Closer inspection of *P. trichocarpa* entries, however, reveals that they are all partial sequences and constitute multiple copies of four laccase proteins.

The AA5 family, comprising 11 members, contains extraplasmic copper radical oxidases of unknown function, with similarity to glyoxal oxidases, and using oxygen as the acceptor to oxidize aldehydes, with the generation of hydrogen peroxide (Daou and Faulds, 2017). The plant members studied so far were implicated in defense (Guan *et al*., 2010), seed coat mucilage cohesion and cell adhesion (Šola *et al*., 2019), and pollen development (Phan *et al*., 2011). The proposed enzymatic activity of one characterized member, RUBY, was the RG‐I galactose oxidase (Šola *et al*., 2019). One of the 11 identified AA5 members has been previously annotated in *P. tomentosa* as glyoxal oxidase in the CAZy database (http://www.cazy.org).

The AA7 family, containing 14 members in *P. trichocarpa*, comprises glucooligosaccharide oxidases (GOOs) that oxidize the anomeric carbon hydroxyl groups of glucose or α‐ and β‐1,4‐linked sugars, first to lactone and then to the corresponding acid, and reduce O_2_ to H_2_O_2_ (Levasseur *et al*., 2013). It includes plant apoplastic proteins named as berberine bridge enzymes and nectarins, which are thought to function in defense responses (van Hellemond *et al*., 2006; Benedetti *et al*., 2018). They have been implicated in the oxidation of oligogalacturonides, functioning as damage‐associated molecular patterns (DAMPs) (Benedetti *et al*., 2018), and possibly in lignin polymerization by the oxidation of monolignols (Daniel *et al*., 2015).

The AA6 family, with six members in *P. trichocarpa*, contains intracellular 1,4‐benzoquinone reductases, which might be involved in detoxification. One member in *A. thaliana*,* AtFRQ1*, was found to be rapidly induced by auxin (Laskowski *et al*., 2002).

#### Carbohydrate binding modules

Carbohydrate binding modules (CBMs) are non‐catalytic domains with autonomous folding that recognize specific carbohydrate motifs (Boraston *et al*., 2004). They can be part of enzymes and receptors or can make an entire protein like OLE E 10 (CBM43), found in the pollen of the olive tree (Barral *et al*., 2005). Among all CBMs, CBM57 was the most abundant in *Populus* (Table 1). This domain, known as the malectin domain in animals, has been implicated in a protein glycosylation surveillance mechanism in the endoplasmic reticulum (ER) (Schallus *et al*., 2008). In plants, CBM57 is found in receptor‐like kinases (RLKs) (Shiu and Bleecker, 2003; Galindo‐Trigo *et al*., 2016). Analysis of sequences of *Populus* proteins containing CBM57 revealed that the majority have a kinase domain and that many are homologous to *Catharantus roseus* RLK1‐like (*Cr*RLK1L) kinases involved in cell wall integrity sensing, polar growth and responses to stresses, such as *At*FERRONIA and *At*HERKULES1 (Engelsdorf and Hamann, 2014). Protein families with CBM57 were expanded in *Populus* compared with *A. thaliana* (Table 1). The evolutionary basis for this expansion is unknown but could be related to more diversified stress reactions in *Populus* as a result of its perennial lifestyle.

Certain CBMs were observed within *Populus* GH families (Tables 1 and S7). The most frequently observed combinations were CBM43–GH17 (callose binding domain–glucan endo‐1,3‐β‐glucosidase), CBM18–GH19 (chitin binding domain–chitinase), CBM48–GH13 (glycogen binding domain–amylase) and CBM22–GH10 (xylan binding domain–xylanase). The only GT with a CBM observed in *Populus* was *Pt*StSy6 from GT5 with three repeats of CBM53, homologous to *A. thaliana* starch synthase III involved in transitory starch biosynthesis (Valdez *et al*., 2008).

The CAZy database contains several entries annotated as CBMs in *Populus* but these lists are incomplete. For example, five out of 15 CBM18‐containing chitinases, one out of eight CBM22‐containing GH10 sequences, eight out of 43 CBM43‐containing endo‐1,3‐β‐glucosidases and nine out of 34 CBM43s containing other proteins were previously identified (http://www.cazy.org; Table 1). Moreover, the smaller CBM families in *Populus*, such as CBM13, 20, 32, 45 and 53, have not been previously annotated in the CAZy database.

### Differential expression of CAZymes in different tissues

Previous CAZyme expression analyses in *Populus* were based on EST frequencies (Geisler‐Lee *et al*., 2006) or microarray analysis (Aspeborg *et al*., 2005). Comparisons of transcriptomes between leaf and developing wood were also made in *P. trichocarpa* using more sensitive RNA sequencing technology (Hefer *et al*., 2015). Here, we used RNA sequencing data sets available for aspen (Sundell *et al*., 2015, 2017; Immanen *et al*., 2016) to perform comparative expression analyses among larger collections of different tissues, which afforded a more sensitive detection of expressed genes. Indeed, normalized expression values showed that of the 1914 CAZyme and expansin gene models, as many as 1796 (94%) were expressed in at least one of the organs and tissues assayed (Table S9). Ten of these genes (0.5%) were found specifically and highly expressed [organ/tissue specificity score of τ = 1 and variance‐stabilizied (VST) expression ≥1.2], suggesting specialized functions for these genes in these organs/tissues (Table 2).

**Table 2:** CAZymes with specific expression pattern in one organ/tissue of aspen and expression value ≥1.2^a^

Potri ID	Pt name	CAZy family	Expression (VST)	Specific in:
Potri.002G202100		GH28	1.27	Expanding flowers
Potri.003G223500		EXPN	1.21	Drought‐stressed leaves
Potri.015G040700		AA1	1.29	
Potri.008G010700		GH28	1.20	Petiole
Potri.016G107900		AA1	1.25	Roots
Potri.001G351600		CBM43	1.24	Mature seeds
Potri.001G223700		GH1	1.24	
Potri.014G082000		EXPN	2.02	Developing phloem
Potri.011G077400	XTH10	GH16	2.10	
Potri.005G054000		CBM18‐EXPN	2.00	Developing xylem

a Expression data from Sundell *et al*. (2015) and Immanen *et al*. (2016). All organs/tissues considered as listed in Table S9.

**Figure 1:**
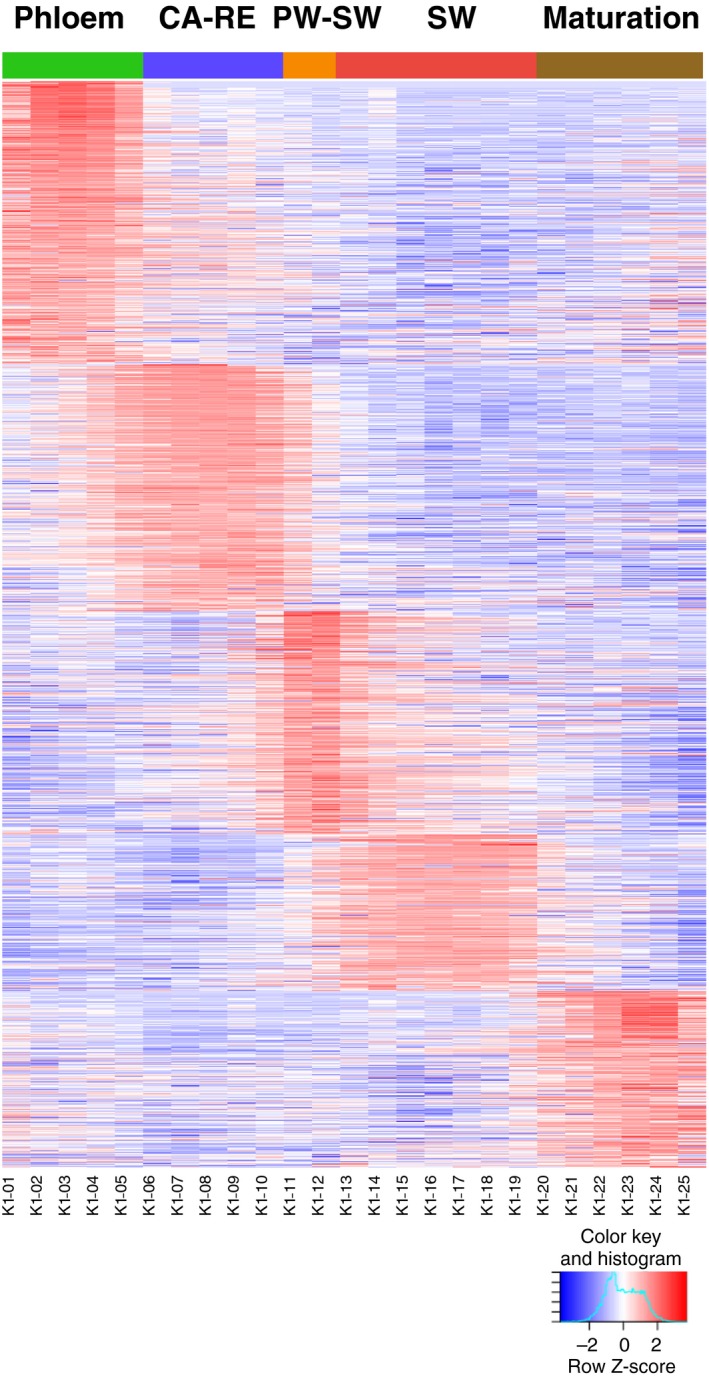
Heat map of CAZyme expression patterns showing that the majority of CAZymes expressed in wood‐forming tissues of aspen (1187 genes) have maximum expression (shown as red color) at specific wood developmental stages, defined by the position (section number) on the bottom. Different developmental zones were defined based on the expression of marker genes (Sundell *et al*., 2017). The expression profiles were sorted by tissue specificity and maximum expression in corresponding tissue. Abbreviations: CA–RE, cambium and radial expansion zone; PW–SW, primary to secondary wall transition; SW, secondary wall formation zone. Data analyzed from AspWood database (http://aspwood.popgenie.org/aspwood-v3.0/).

To functionally characterize CAZymes during wood biosynthesis, we examined their expression pattern in the AspWood database (http://aspwood.popgenie.org/aspwood-v3.0/), which represents a high‐spatial‐resolution transcript analysis of developing aspen wood (Sundell *et al*., 2017). A total of 1187 CAZyme models (62%) were found expressed in wood‐forming tissues (Table S10), the majority of which exhibited defined patterns of expression in developing wood, peaking at different stages of wood formation (Figure 1). These patterns indicate that certain sets of CAZymes have specific functions during wood development, which are separated along the pseudotime developmental program, assayed from the cambium to mature xylem, within these cryosection series. The phloem tissue cluster (as defined in Figure 1) contained the largest number of CAZyme genes, which were linked to cell wall biosynthesis, defense or phloem assimilate transport, as expected. The second largest cluster was associated with the cambium–radial expansion zone, reflecting the diverse metabolic activities required for intense primary cell wall biosynthesis and modification. The primary to secondary wall transition zone was another developmental zone where a large number of CAZymes peaked, most likely in connection with the reorganization of the cell wall biosynthetic machinery.

Subsequent analysis of each cluster composition with regards to different CAZyme families revealed remarkable variation among different wood developmental zones, with different groups of CAZymes dominating in these zones (Figure S2; Table S11). The phloem cluster contained the highest share of CMBs, the cambium–radial expansion zone was characterized by the highest proportion of expansins and PLs, the primary to secondary wall transition zone had the highest proportion of AAs and CEs, the secondary wall formation zone had the highest proportion of GTs and the maturation zone had the highest proportion of GHs (Figure S2; Table S11). Among the GTs, GT1 and GT4 (sucrose synthase) dominated in the phloem, GT2 (largely represented by CSLD genes), GT31, GT57, GT66 and GT75 (UDP‐arabinose mutases) had highest representation in the cambium and radial expansion zone, GT8 (represented mainly by galacturonyl transferases involved in pectin and xylan biosynthesis) dominated during the primary to secondary wall biosynthesis transition and GT43 (involved in xylan biosynthesis) was most prominently represented in the secondary wall formation zone (Figure S2; Table S11). Within the GHs, phloem had the highest share of GH1, GH5 (GH5_11 and GH5_14) and GH14 (β‐amylases), the cambium and radial expansion zone showed the highest abundance of GH16 (XTH), GH9 (cellulases) and GH36, the primary to secondary wall transition zone was most abundant in GH3, GH38 and GH47, the secondary wall formation zone was most abundant in GH28 (polygalacturonases) and the maturation zone ‐ in GH17 (β‐1,3,‐glucanases) (Figure S2; Table S11). This highlights the diversity of CAZyme functions during different stages of xylogenesis.

The cluster analysis (Figure S2; Table S11) provided some insights into the role of the recently annotated CAZyme gene families in *Populus*. For example, GH152 was abundant in the phloem and cambium–radial expansion zones, consistent with its proposed role in callose metabolism (Trudel *et al*., 1998; Sakamoto *et al*., 2006). CBM57 (malectin domain‐receptor‐like kinases) was also most abundantly represented in the same tissues, consistent with the suggested roles in signaling and stress responses (Engelsdorf and Hamann, 2014). Most CE6 members were expressed in the secondary wall formation zone and the AA6 family was most abundant at the primary to secondary transition, which supports the roles of these families in secondary wall biosynthesis. GT106 (Takenaka *et al*., 2018) had numerous members from different clades reaching high abundance in several xylem cell developmental zones, pointing to their importance at different stages of xylogenesis.

### Classification of CAZymes into functional groups related to starch and cell wall biosynthesis

We classified *Populus* CAZymes into different metabolic groups based on their functional and biochemical characterization or their similarity to enzymes characterized in other plant species (Table S12). Furthermore, as most biochemically and functionally characterized genes were from *A. thaliana*, we determined their likely orthologs in *Populus* by phylogenetic analysis, and in some cases proposed their names based on the well‐studied *A. thaliana* genes (Figure S1; Tables S1–S7). This approach assumes functional conservation for well‐defined clades and although this is generally a valid assumption, there could be exceptional cases of neofunctionalization in one species, especially when linked to disproportionate clade expansion. The clade information as well as highest sequence homology score data are included in the Table S12. The functional categories were broadly classified as starch and sugar related (sucrose, raffinose, stachyose, galactinol and trehalose), cell wall polymer related, including AGPs/extensins, callose, cellulose, xylan, mannan, pectin, xyloglucan and lignin‐related categories. Some enzymes that have a role in more than one type of polymer biosynthesis/modification were separately classified as pectin/xylan, etc. Genes belonging to the same families as the characterized CAZymes but to different clades were classified as ‘uncharacterized’ (Table S12).

### Variation of CAZyme transcriptome related to different cell wall polymers during wood formation reflects their biosynthesis pattern

**Figure 2:**
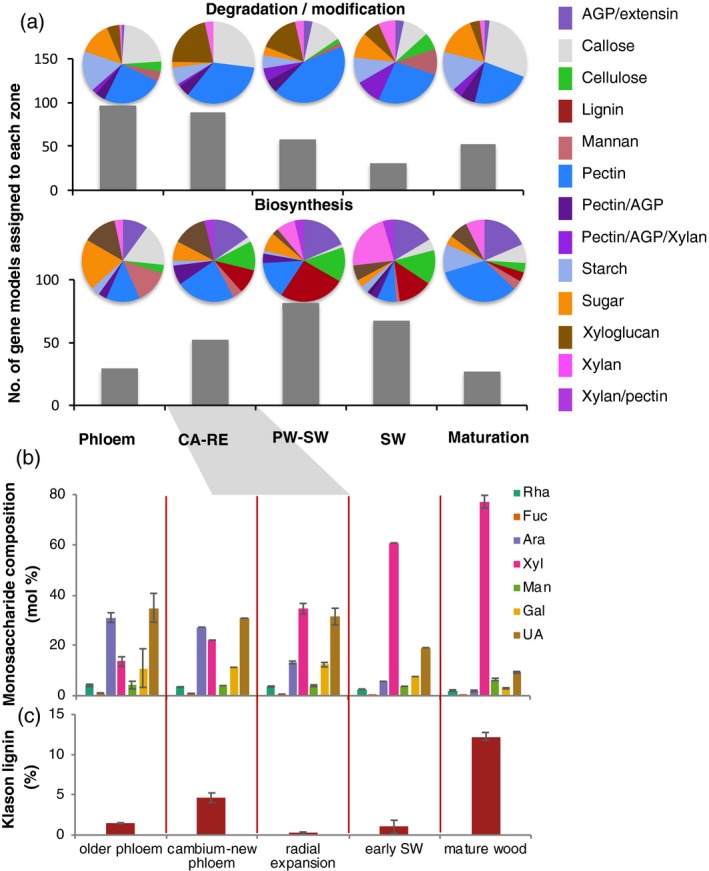
Variability in cell wall composition and in the CAZyme transcriptome related to the biosynthesis and degradation of different polymers across wood developmental zones. (a) Count of gene models associated with specific wood developmental zones (bar graph) and the composition of each group according to predicted functions (pie charts). Data based on Sundell *et al*. (2017). Gene metabolic classification as listed in Table S12. ‘Sugar’ category includes enzymes related to sucrose, raffinose, stachyose, galactinol and trehalose metabolism. Abbreviations: CA–RE, cambium–radial expansion zone; PW–SW, primary to secondary wall transition; SW, secondary wall formation zone. (b and c) Analysis of cell wall composition in different samples of developing secondary phloem and xylem by alditol acetates (excluding Glc) and uronic acid (UA) contents (b) and Klason lignin content (c). Cell wall samples were obtained by the sequential scraping of bark and wood surfaces exposed after peeling the bark. Alignment between transcriptome and cell wall samplings is shown by the gray shadow. Data in (b) and (c) are mean values from *n *= 3 technical replicates ± SE.

To provide support for our functional annotations, we subsequently analyzed the composition of transcriptomes related to the biosynthesis and degradation of different cell wall components in different zones of developing wood, sampled at high spatial resolution (Sundell *et al*., 2017), and compared this with changes in cell wall sugar composition and in lignin content during wood development. The CAZymes were clustered according to their maximal expression values as preferentially expressed in different developmental zones, shown in Figure 1, and the number of genes categorized into different metabolic activities were compared among these different clusters. The transcriptome analysis revealed distinct variability across the wood developing zones in both the number of genes with highest expression in a particular zone and in their metabolic functions (Figure 2a; Table S13). Polysaccharide degradation/modification‐related genes were mostly clustered in the phloem and cambium–radial expansion zones. The phloem cluster was relatively rich in starch and sugar degradation‐related genes, whereas in the cambium–radial expansion zone, xyloglucan degradation/modification‐related genes dominated. The primary to secondary wall transition zone was especially abundant in pectin degradation/modification‐related genes, the secondary wall zone was abundant in mannan degradation‐related genes, whereas the maturation zone transcriptome, corresponding mostly to the live xylem parenchyma cells, had the largest share of callose degradation‐related genes. Biosynthesis‐related genes were most abundant in the primary to secondary wall transition zone (Figure 2a). Phloem was enriched in sugar, callose and mannan biosynthetic genes, the cambium–radial expansion zone was enriched in pectin biosynthesis‐related genes, the primary to secondary wall transition zone had the largest share of lignin‐biosynthetic CAZy genes, and the secondary wall zone was enriched in xylan‐related genes. The maturation zone, where mostly parenchyma cells contributed to the transcriptome, was enriched in pectin and starch biosynthesis‐related genes. Pectin constitutes the main part of the protective layer formed by parenchyma cells after secondary wall deposition (Mellerowicz *et al*., 2001).

To reveal whether the observed changes in the transcriptome corresponded to cell wall composition, samples across the developmental gradient of secondary phloem and secondary xylem were obtained by splitting the bark and wood in actively growing aspen trees and sequentially scraping the tissues on both exposed sides. The monosaccharide composition of trifluoroacetic acid (TFA)‐ hydrolyzed polymers and lignin were analyzed in these samples. These samples were aligned with the transcriptome series (shown by gray shading in Figure 2b,c) based on the bark‐splitting site in the radial expansion zone (Gray‐Mitsumune *et al*., 2004). A shift in monosaccharide composition between primary (radial expansion and outward samples) and secondary (early SW and mature wood) walled samples was clearly observed (Figure 2b), with the primary walled samples having a high content of pectin‐related sugars, arabinose and uronic acids, whereas the secondary walled xylem samples were dominated by xylose, reflecting their high xylan content. The mannose content was relatively constant in all samples. These data agree with published knowledge on the composition of primary and secondary wall layers in hardwoods (Mellerowicz *et al*., 2001; Mellerowicz and Gorshkova, 2012) and with observed transcriptome variability regarding the pectin and xylan biosynthesis‐related genes (Figure 2a).

**Figure 3:**
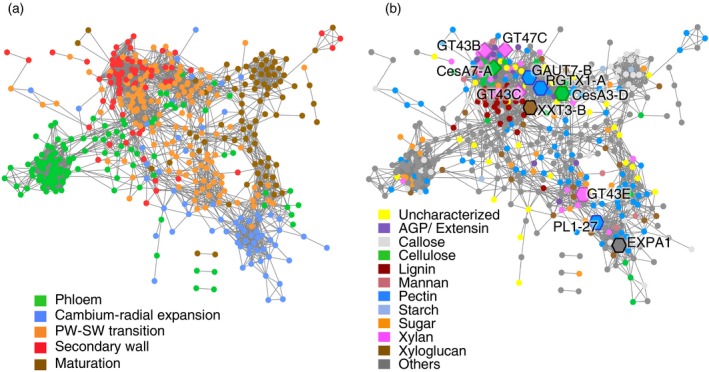
The co‐expression networks of CAZymes in wood‐forming aspen tissues (http://aspwood.popgenie.org/aspwood-v3.0/). The CAZyme gene co‐expression at a context likelihood of relatedness threshold of 5 or higher was extracted as a network from popgenie (http://popgenie.org/exnet) and visualized in cytoscape 3.4.0. (a) The genes colored by developmental zones where they have peak expression as shown in Fig. 1. (b) The same network colored by the CAZyme metabolic function. ‘Uncharacterized’ class includes members of families with probable function in cell wall biosynthesis. The ‘Sugar’ category includes enzymes related to sucrose, raffinose, stachyose, galactinol and trehalose metabolism. Lists of all genes in the networks are given in Table S14. The guide genes used in the primary wall forming zone are marked by hexagons and those of the secondary wall are marked by diamonds. The first neighbors of the guide genes are listed in Tables S15 and S16.

The lignin content showed a small peak in the young phloem sample and it increased largely in mature xylem (Figure 3c). The phloem peak identifies the zone of phloem fiber differentiation and is supported by transcript data for lignin‐ and many secondary wall‐related genes (Sundell *et al*., 2017).

### Uncharacterized CAZymes involved in wood biosynthesis: construction of CAZyme‐based co‐expression networks in wood‐forming tissues

The co‐expression of uncharacterized CAZyme genes with the already known genes in wood‐forming tissues may reveal novel genes involved in wood formation. A total of 471 CAZyme genes formed a co‐expression network, which included many uncharacterized genes with putative functions in cell wall biosynthesis and in other cellular processes during wood formation (Table S14). We have colored the genes in the network by their expression cluster (Figure 3a) and by their metabolic function (Figure 3b). The network clearly illustrates the existence of subnetworks related to different stages of xylem cell differentiation (Figure 3a) and, in a few cases, related to the metabolism of different compounds (Figure 3b). The metabolism of different compounds can be illustrated by the separation of lignin‐ and carbohydrate‐related networks during secondary wall formation, suggesting that these metabolic processes have distinct spatiotemporal regulation.

To identify more candidates for wood cell wall biosynthesis and modification, we have analyzed the closest neighbors of some known cell wall‐related CAZymes using a ‘guide genes’ approach (Aoki *et al*., 2007) separately for the primary wall‐ and secondary wall‐related networks (Tables S15 and S16, respectively).

#### Primary wall CAZyme networks

Within the primary wall network, we focused on the first neighbors of the cellulose, pectin and xyloglucan‐related genes that were used as ‘guides’ (Figure 3; Table S15), as the primary wall of aspen xylem contains mostly these components (Mellerowicz *et al*., 2001). The guide genes included the primary wall CesA *PtCesA3‐D* (Kumar *et al*., 2009), the *Populus* ortholog of *AtGAUT7* encoding homogalacturonan α‐1,4‐galacturonosyl transferase (Atmodjo *et al*., 2011) *PtGAUT7-B*, the *Populus* ortholog of *AtRGXT1* encoding RG‐II‐α‐1,3‐xylosyl transferase (Egelund *et al*., 2006; Liu *et al*., 2011) *PtRGXT1-A*, and the *Populus* ortholog of *AtXXT3* encoding a candidate xyloglucan α‐1,6‐xylosyl transferase (Vuttipongchaikij *et al*., 2012) *PtXXT3-B/PtGT34B*. The obtained cellulose–pectin–xyloglucan network (Table S15) included several putative xyloglucan and pectin biosynthesis‐related genes, as identified by the phylogenetic analysis (Figure S1), such as *PtXXT1‐A*,* PtGAUT9‐A*,* PtGAUT13‐A*,* GATL11‐B*, two uncharacterized GT47 members from the clade of *AtARAD1* involved in pectic arabinan biosynthesis (Potri.T071700 and Potri.019G086800; Harholt *et al*., 2012), and putative polygalacturonases from GH28 (Potri.016G051200 and Potri.016G054800) (Table S15). Interestingly, the primary wall cellulose–pectin–xyloglucan network included two GT106 members (Table S15), one from the RGI‐I:rhamnosyltransferase (RRT) clade (Potri.015G048100; Takenaka *et al*., 2018) and another (Potri.003G062600) from a different clade that has not yet been characterized (Figure S1), indicating the involvement of these GT106 members in xylem primary wall biosynthesis. The network also included a GT29 member Potri.014G145400 (Table S15), similar to *At*SIA1/*At*MGP2 and *At*SIA2 transferases that have been proposed to be involved in transferring of DHA or KDO to the RG‐II backbone (Deng *et al*., 2010; Dumont *et al*., 2014). Another co‐expressed gene was Potri.019G082200 from GT10. In *A. thaliana*, Golgi localized GT10 α‐1,3‐or α‐1,4‐fucosyltransferases have been proposed to function in *N*‐glycan biosynthesis (Both *et al*., 2011; Rips *et al*., 2017). Our data suggest a role for the GT10 family in primary cell wall biosynthesis in the xylem. Other genes of this network belong to families GT14, GT31 and GT77, with putative functions in primary wall extensins and AGP biosynthesis (Gille *et al*., 2013; Knoch *et al*., 2013; Basu *et al*., 2015) (Table S15).

The network of the primary wall xylan biosynthetic gene *PtGT43E* (Ratke *et al*., 2015, 2018) was distinct from that of the cellulose–pectin–xyloglucan biosynthesis network, and shared some genes with the network of α‐expansin *Pt* *EXPA1* (Gray‐Mitsumune *et al*., 2008) and pectate lyase *Pt* *PL1‐27* (Biswal *et al*., 2014), the key expansion markers for xylem cells (Sundell *et al*., 2017) (Table S15; Figure 3B). This network included additional expansin and *PL1* genes, *XTH*,* PL4* members, xylan degradation‐related genes *PtXYN10E* and *PtBXYL2*, a cellulase‐encoding gene *PtGH9B3* (Takahashi *et al*., 2009) and a putative mannanase‐encoding gene *PtMAN7* (Table S15), which are all likely to be involved in xylem cell expansion. This extends the list of known cell wall modifying enzymes with putative function in xylem cell expansion, and suggests that all classes of polymers are modified during this process. Interestingly, the network also included three members of the AA1 family of the SKU clade that has not yet been characterized in the context of wood development (Figure S1; Table S15). The clade founding the *AtSKU5* gene has been implicated in directional growth of roots (Sedbrook *et al*., 2002), suggesting a role for these *Populus* SKU5‐like members in wood growth.

#### Secondary wall CAZyme networks

The secondary wall CAZyme networks included many genes of proven or putative function in the metabolism of xylan, AGP/extensin, cellulose, mannan, lignin and pectin (Figure 3; Table S16). To identify novel candidate genes involved in secondary wall biosynthesis, we analyzed first‐order neighbors of genes that have been shown to be involved in the biosynthesis of: secondary wall cellulose – *PtCesA7‐A* (Kumar *et al*., 2009; Song *et al*., 2010); secondary wall xylan – *PtGT43B* and *PtGT43C*, involved in xylan backbone biosynthesis (Lee *et al*., 2011; Ratke *et al*., 2018); and *PtGT47C*, a homolog of *AtFRA8*, involved in the reducing end sequence biosynthesis in xylan (Lee *et al*., 2009). The neighbors of *PtCesA7‐A*,* PtGT43B* and *PtGT47C* largely overlapped and included several known genes encoding the components of the secondary wall cellulose synthase complex, such as CesAs (*Pt*CesA4, *Pt*CesA7‐A, *Pt*CesA8‐A and *Pt*CesA8‐B; Song *et al*., 2010), cellulase *Pt*Cel9A1/*Pt*KOR1, which is required for cellulose biosynthesis (Nicol *et al*., 1998; Szyjanowicz *et al*., 2004) and cellulose crystallinity regulation (Takahashi *et al*., 2009; Maloney and Mansfield, 2010), *PtGH19A* ‐ the homolog of *At* *CTL/* *At* *POM1/* *At* *ELP1*, which is essential for cellulose biosynthesis but of unknown molecular function (Sánchez‐Rodríguez *et al*., 2012), and the sucrose synthase *Pt*SUS2 (Gerber *et al*., 2014). All these proteins except *Pt*GH19A were found to be co‐immunoprecipitated with secondary wall CesAs in *Populus*, suggesting their connection to the cellulose synthase complex (Song *et al*., 2010) and supporting the rationale of using the network analysis for finding additional components of secondary wall biosynthesis. Moreover, we have identified most of the genes with proven or proposed function in secondary wall xylan biosynthesis (Rennie and Scheller, 2014). These included the genes encoding proteins of the xylan synthase complex (Jiang *et al*., 2016; Zeng *et al*., 2016): the pair of orthologs to *AtIRX10* (Jensen *et al*., 2014), *PtGT47A-1* and *PtGT47A-2*, one of the pair of orthologs to *AtIRX10L* (Wu *et al*., 2009; Mortimer *et al*., 2015) *PtGT47D-1* (Figure S1; Table S12), and *AtIRX14* clade member *PtGT43D* (Ratke *et al*., 2015, 2018) (Table S16). Thus, similar to Arabidopsis, the xylan:xylosyl transferase activities in secondary walled xylem cells in poplar appear to be redundantly encoded by homologs to *AtIRX10* and *AtIRX10L* (Wu *et al*., 2009). Furthermore, we identified genes encoding enzymes involved in xylan reducing end biosynthesis in the secondary wall network: *PtGAUT12‐A/PtGT8D‐1* and *PtGAUT12‐B/PtGT8D‐2* (Li *et al*., 2011b; Biswal *et al*., 2015), a pair orthologous to *AtIRX8* (Persson *et al*., 2007); and *PtGATL1‐A* and *PtGATL1‐B*, a pair orthologous to *AtPARVUS* (Kong *et al*., 2009) (Figure S1; Table S12). The network also included genes encoding putative glucuronosyl transferases, *PtGUX1‐A* and *PtGUX1‐B*, a pair orthologous to *AtGUX1* (Figure S1; Table S12), and known to be involved in the generation of GlcA decorations on the xylan backbone with an even spacing pattern, forming the major xylan domain of secondary walls (Mortimer *et al*., 2010; Bromley *et al*., 2013). Xylan transglycosylase *Pt*XYN10A (Derba‐Maceluch *et al*., 2015) was also a part of the secondary wall CAZyme network.

Mannan‐related genes found in the secondary wall network included the glucomannan synthase encoding gene *PtCSLA1/PtGT2A* (Suzuki *et al*., 2006) and the putative endo‐mannanase gene *PtMAN4* (Yuan *et al*., 2007; Zhao *et al*., 2013a).

The uncharacterized candidates among the CAZymes identified in the secondary wall network included the CE6 member Potri.014G022600 with putative xylan acetyl esterase function, three members of GT106, one of which was from the clade of RG‐I:rhamnosyl transferases (Takenaka *et al*., 2018), putative pectin modifying genes from families CE13, PL4 and GH28, and putative AGP synthesizing members of the GT14 and GT31 families (Table S16). The network also included nine AA1 genes belonging to the laccase clade (Figure S1), homologous to *AtLAC4/AtIRX12*,* AtLAC11*,* AtLAC13* and *AtLAC17*, of which *AtLAC4*,* ‐11* and *‐17* are known to function in xylem cell lignification (Berthet *et al*., 2011; Zhao *et al*., 2013b).

Although several CAZymes known to be involved in secondary wall xylan biosynthesis were co‐expressed as close neighbors of secondary wall CesAs, *PtGT43C* neighbors did not include secondary wall CesAs except *PtCesA8‐A,* and did not extensively overlap with the secondary wall xylan network (Figure 3B; Table S16). Instead, this subnetwork included some members of the primary wall CAZyme network, such as two GT106 genes, a putative xyloglucan xylosyl transferase *PtXXT3‐B/PtGT34B*, a putative homogalacturonan synthase *PtGAUT7‐B*, and two uncharacterized GT47 members similar to *AtARAD1* (Tables S15 and S16). This agrees with a proposal that *Pt*GT43C participates in both the primary and the secondary wall xylan biosynthetic complexes (Ratke *et al*., 2015, 2018; Sundell *et al*., 2017). Thus, the *PtGT43C* subnetwork is expected to include other genes with functions in primary and secondary walls. We identified several genes with putative AGP/extensin biosynthesis functions from families GT14 and GT31 in this subnetwork, as well as genes from uncharacterized CAZyme families: for example, AA6 or GH47 (Table S16).

### Associations of CAZymes with other genes involved in wood secondary wall biosynthesis

To identify more co‐expressed genes and their regulators involved in secondary wall biosynthesis in developing xylem, we extended the co‐expression analyses to all genes of *P. trichocarpa*, using the same stringency (threshold 5) as in CAZyme networks. Cellulose, xylan and glucomannan are the main polysaccharides of secondary wall layers in aspen, and hence we used CAZyme genes involved in the biosynthesis of these polymers as guide genes, including the prominent members of the identified CAZyme network associated with secondary wall biosynthesis (Table S16). By using this approach, we identified 354 gene models co‐expressed with one or more of secondary wall guide genes (Table S17).

As expected, the network included the previously identified CAZymes with known or putative functions in secondary wall cellulose, xylan and glucomannan biosynthesis. In aspen wood, all matrix polysaccharides are acetylated, which affects their water solubility, ability to undergo enzymatic modification of degradation, and interactions with cellulose and other polymers (Pawar *et al*., 2013; Busse‐Wicher *et al*., 2014). Accordingly, the acetylation‐related genes were a prominent group in the secondary wall network. They included *PtRWA‐A* (Pawar *et al*., 2017), several members of the TBL family of acetyltransferases homologous to *AtTBL3*, ‐*29*, ‐*33* and ‐*34* (Xiong *et al*., 2013; Yuan *et al*., 2013, 2016a,2016b,2016c; Urbanowicz *et al*., 2014), as well as *AtAXY9* (Schultink *et al*., 2015) homolog Potri.017G139800 (Table S17, cf. acetylation category).

**Figure 4:**
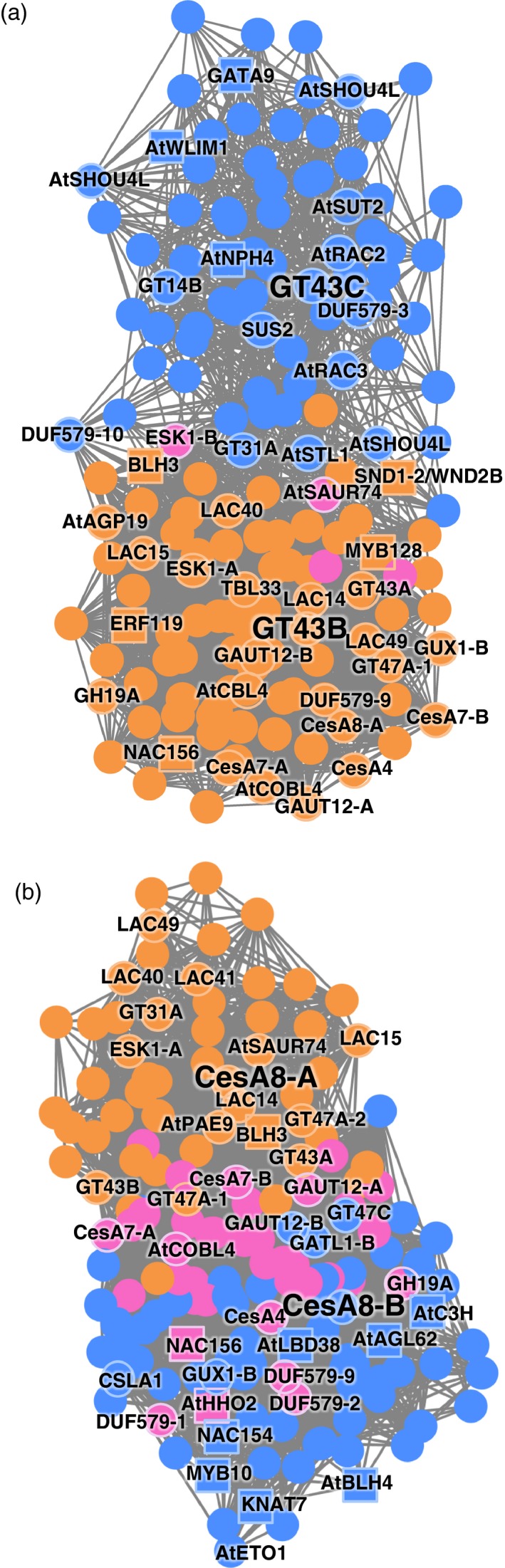
The difference in neighborhoods of genes encoding proteins of secondary wall xylan synthase complex *Pt* GT43B and *Pt* GT43C (a), and secondary wall cellulose synthase complex *Pt*CesA8‐A and *Pt*CesA8‐B (b). The co‐expressed genes were selected at threshold = 5 (http://aspwood.popgenie.org/aspwood-v3.0/). The blue nodes are unique neighbors of *Pt* GT43C (in a) and *Pt*CesA8‐B (in b) and the orange nodes are unique for *Pt* GT43B (in a) and *Pt*CesA8‐A (in b). The pink nodes are neighbors in common for both genes in (a) and (b). The transcription factors are marked by rectangles. Unless otherwise indicated, the *Populus* gene names are used in the figures.

The glucuronic acid side chain of xylan in woody species, including aspen, is methylated at *O*‐4 (Teleman *et al*., 2000), and this methylation is essential for wood growth and secondary wall formation (Song *et al*., 2014). It is generated in the Golgi with the help of glucuronoxylan methyl transferases (GXMTs) found in clade A of the DUF579 family (Lee *et al*., 2012; Urbanowicz *et al*., 2012). So far, GXMT activity has been identified in four members of the *Populus* DUF579 gene family, *Pt*GXMT1/*Pt*DUF579‐2, *Pt*GXMT2/*Pt*DUF579‐1, *Pt*GXMT3/*Pt*DUF579‐3 and *Pt*GXMT4/*Pt*DUF579‐4 (Song *et al*., 2014; Yuan *et al*., 2014), with the first three being part of the secondary wall network (Table S17, cf. DUF579 category). Clade B of the DUF579 family contains a pair of *AtIRX15* and *AtIRX15‐L* genes that are required for glucuronoxylan biosynthesis in secondary cell walls (Brown *et al*., 2011; Jensen *et al*., 2011), their orthologous pair *PtDUF579‐10* and *PtDUF579‐9* (Song *et al*., 2014) were also a part of the secondary wall network (Table S17, cf. DUF579 category). Interestingly, *PtDUF579‐9* is expressed with *PtGH43B*, whereas *PtDUF579‐10* is expressed as a first neighbor of *PtGT43C* (Table S17). These two expression networks formed separate clusters with very little overlap (Figure 4a), and we propose that the former is associated strictly with the secondary wall formation and the latter includes genes acting at both primary and secondary wall formation stages (Ratke *et al*., 2015, 2018).

The fasciclin‐like arabinogalactan proteins (FLAs) play an important role in cell wall architecture in *A. thaliana* and *Populus*, mediating wall mechanical properties (Johnson *et al*., 2003; Andersson‐Gunnerås *et al*., 2006; Wang *et al*., 2015). We observed several homologs of *AtFLA17* and *AtFLA11* in the secondary wall network (Table S17, cf. AGP category). Their presence, along with the presence of a lysine‐rich arabinogalactan protein similar to *At*AGP19, is matched with the abundance of CAZymes with proposed function in AGP glycosylation (Table S16).

The secondary wall network had a prominent representation of proteins involved in vesicle formation and movement, and in rosette movement (Table S17, cf. vesicle, movement category). The identified genes are good candidates for the biotechnological regulation of secondary wall formation, as the regulation of vesicle and cellulose synthase complex movement is likely to limit the biosynthesis of the secondary wall (McFarlane *et al*., 2014; Wang *et al*., 2016).

Another very prominent group of the secondary wall network were the signaling‐ and protein modification‐related genes, including genes encoding receptor‐like kinases with a lectin domain (Table S17, cf. ‘signaling’ category). Several of these proteins are known as receptors involved in cell wall integrity (CWI) signaling that perceive cell wall damage and activate diverse responses, including gene expression (Engelsdorf and Hamann, 2014). CWI was described in primary walled cells, but whether equivalent CWI signaling exists in cells depositing secondary walls is currently under debate because no expected changes in gene expression were detected in *A. thaliana* mutants affected in secondary wall xylan biosynthesis (Faria‐Blanc *et al*., 2018). The mutants in secondary wall CesA (Hernandez‐Blanco *et al*., 2007) and secondary wall acetylation (Pawar *et al*., 2016) exhibited altered expression of resistance genes, however, as well as increased resistance to pathogens, which were assumed to be triggered by CWI. Moreover, Ratke *et al*. (2018) reported altered gene expression and increased growth in aspen with reduced secondary wall xylan biosynthetic GT43 genes, and suggested that these changes were mediated by CWI in secondary walled cells. Our finding of several receptor‐like kinases in the secondary wall network (Table S17) provides strong candidates for secondary CWI sensing. Interestingly, the dominant repressors of the double mutant *fei1 fei2* (impaired in CWI) have been recently identified as *SHOU4* and *SHOU4L* in *A. thaliana* (Polko *et al*., 2018), and the secondary wall network contained three homologs of these genes associated with *PtGT43C* (Figure 4a; Table S17, cf. cell wall category), strengthening the hypothesis of the secondary CWI pathway.

More attention has been given to the transcription factors regulating the onset of secondary wall development (Ohtani *et al*., 2011; Zhong *et al*., 2011; Taylor‐Teeples *et al*., 2015), as they can offer direct genetic approaches to improve the productivity of trees. We have identified several well‐known transcription factors as co‐expressed neighbors with different secondary wall biosynthetic CAZyme genes (Table 3). As we observed clear differences in networks of *PtGT43C* and *PtGT43B*, confirming the previous conclusions that *PtGT43C* functions in both primary and secondary xylan biosynthesis, whereas *PtGT43B* is specifically involved in secondary wall xylan biosynthesis (Ratke *et al*., 2015, 2018), we analyzed the transcription factors in these networks in order to reveal the nature of their regulons (Figure 4a). The secondary wall‐associated NAC domain (SND) and vascular‐related NAC domain (VND) proteins are known as master switchers of secondary wall formation in *Populus* (Ohtani *et al*., 2011; Zhong *et al*., 2011; Li *et al*., 2012). The overexpression of *PtSND1‐B2/PtWND2B* resulted in the ectopic deposition of secondary walls along with enhanced expression of the genes involved in the biosynthesis of wood components, cellulose, xylan and lignin (Zhong *et al*., 2011; Li *et al*., 2012). We identified *PtSND1‐B2/PtWND2B* co‐expressed as a close neighbor of *PtGT43B* (Figure 4a; Table 3), in agreement with the previous reports of *Pt*SND1‐B2/*Pt*WND2B activating the *PtGT43B* promoter (Ratke *et al*., 2015). Some of the important primary targets of *Pt*SND1‐ B2/*Pt*WND2B, such as *PtKNAT7*,* PtNAC156*,* PtBLH3*,* PtMYB128* and *PtMYB10* (Zhong *et al*., 2011), have also been found associated with secondary wall CAZymes (Table 3). Of these, *PtNAC156*,* PtBLH3* and *PtMYB128* were associated with *PtGT43B*, but not with *PtGT43C* (Figure 4a). *PtGT43B* was also uniquely co‐expressed with an ethylene response factor (ERF) transcription factor *PtERF119* homologous to *AtSHINE3* (*AtSHN3*), which was recently found as an important hub transcription factor in secondary wall forming aspen xylem (Seyfferth *et al*., 2018). Overexpression of the closely related *Populus SHN2/ERF118* gene induced cellulose and hemicellulose and suppressed lignin biosynthesis in *Nicotiana tabacum* (tobacco; Liu *et al*., 2017). The *PtGT43C* network included genes encoding transcription factors that have not been studied in the context of secondary growth, such as homologs of the GATA family gene *AtGATA9* and an ARF family gene *AtARF7* implicated in auxin‐induced lateral root growth and tropism responses (Goh *et al*., 2012). These and other transcription factors of unknown function found in our analysis (Table 3) could represent targets for tree improvement.

**Table 3:** Transcription factors co‐regulated with genes encoding enzymes synthesizing cellulose, mannan and xylan in secondary walls in the developing wood of aspen (AspWood, http://aspwood.popgenie.org/aspwood-v3.0/)

Potri. ID	Potri name	AT ID	AT name	TF class	CesA7‐A Potri.006G181900	CesA8‐A Potri.011G069600	CesA8‐B Potri.004G059600	GT43B Potri.016G086400	GT43C Potri.007G047500	GT47A‐1 Potri.001G068100	GATL1‐B Potri.002G132900	GATL1‐A Potri.014G040300	GT47C Potri.009G006500	CSLA1 Potri.008G026400	GUX1‐A Potri.007G107200	GUX1‐B Potri.005G061600
001G099800	MYB10	1G63910	MYB103	MYB	+		+						+			
001G112200	KNAT7	1G62990	IXR11, KNAT7	TALE	+		+						+			
001G258400				C3H	+		+			+	+					+
002G119400		3G49940	LBD38	LBD	+		+			+	+					+
002G257800		4G28610	PHR1	G2‐like	+											
006G253800		5G25390	SHN3	ERF	+			+		+	+					+
007G135300	NAC156	4G28500	ANAC073, NAC073, SND2	NAC	+	+	+	+		+	+	+				+
008G117500		1G68670	HHO2	G2‐like	+	+	+									+
009G045900		5G60440	AGL62	M‐type_MADS	+		+			+	+	+				+
017G016700	NAC154	4G28500	ANAC073, NAC073, SND2	NAC	+		+				+		+	+		+
002G031000	BLH3	2G16400	BLH7	TALE		+		+								
002G178700	WND2B, SND1‐B2	2G46770	ANAC043, EMB2301, NST1	NAC				+								
003G132000	MYB128	1G63910	MYB103	MYB				+								
006G138500		5G20730	ARF7,BIP,IAA21,‐23,‐25, MSG1,NPH4,TIR5	ARF					–							
008G038900		4G32890	GATA9	GATA					+							
001G141100		5G46690	bHLH071	bHLH							+					
012G055700		1G73830	BEE3	bHLH												+
005G221400		2G42410	ZFP11	C2H2											+	

+, positive; –, negative correlation

We also noted subtle differences between the CAZyme networks of *PtCesA8A* and those of other secondary wall CesAs. In *A. thaliana*,* AtCesA4*, ‐*7* and ‐*8* are found to be co‐expressed, as their encoded proteins form the secondary wall cellulose synthase complex in equimolar ratios (Hill *et al*., 2014). The situation is more complex in *Populus*, because there are two paralogs of each *AtCesA7* and *AtCesA8* (Kumar *et al*., 2009), creating the possibility of neofunctionalization for one of these duplicated members. We suspected that this could be the case for the *PtCes8‐A/B* pair as their expression in aspen wood‐forming tissues diverged during the later stages of xylogenesis (Sundell *et al*., 2017). The co‐expressed neighbors between *PtCesA8‐A* and *PtCesA8‐B* had significant similarity for cellulose biosynthetic proteins (for example, *PtCesA4*,* PtCes7‐A*,* PtCes7‐B* and *PtGH19A*), but showed differences for transcription factors and signaling‐related proteins (Figure 4b; Tables 3 and S17). They both shared *PtNAC156* (Zhong *et al*., 2011) and Potri.008G117500, a MYB‐like gene with unknown function homologous to *AtHHO2*, controlling phosphate homeostasis (Nagarajan *et al*., 2016), but *PtCesA8‐A* was uniquely associated with *PtBLH3*, a transcription factor downstream of *Pt*SND1‐B2/*Pt*WND2B (Zhong *et al*., 2011), and a SAUR‐like gene, suggesting the involvement of IAA in the regulation of this network. The *PtCesA8‐A* network also included secondary xylan biosynthetic genes strictly expressed during secondary wall formation, *PtGT43A* and *PtGT43B* (Ratke *et al*., 2015), suggesting a secondary wall function for *PtCesA8‐A* (Figure 4b). The unique neighbors of *PtCesA8‐B* included other secondary wall‐controlling transcription factors, including *PtKNAT7*,* PtMYB10* and *PtNAC154* (Zhong *et al*., 2011), as well as a MADS‐box gene homologous to *AGAMOUS LIKE 62* (*AtAGL62*), which is known to regulate the cellularization of the endosperm in *A. thaliana* (Kang *et al*., 2008) (Figure 4b; Table 3). The presence of the *AtETO1* homolog in this subnetwork suggests its negative correlation with ethylene signaling; *At*ETO1 is known to target ACC synthases that are rate‐limiting enzymes in ethylene biosynthesis for proteolysis (Christians *et al*., 2009). Taken together, these observed differences in the neighborhoods of *PtCes8‐A* and *PtCesA8‐B* suggest some additional function(s) for *PtCesA8‐B* in wood development beyond a core secondary wall CesA function. Indeed, *PtCesA8‐B* was found to be the dominant CesA isoform expressed in tension wood of aspen, possibly even forming homomeric cellulose synthase complexes in this tissue (Zhang *et al*., 2018).

## Conclusion

This study provides a census of all CAZymes and expansin‐related proteins in *P. trichocarpa –* a model hardwood species *–* based on the re‐annotation of gene models of the v3.0 genome assembly. The abundance and diversity of CAZymes in *P. trichocarpa* is compared with those in *A. thaliana* revealing no differences in the presence of CAZyme families, but considerable differences in the relative size of certain families, suggesting their adaptive regulation. We report 101 CAZyme families, 18 of which have not been previously annotated in *P. trichocarpa*, and the updated information on family members with 1914 genes in total annotated as CAZymes, which is a major update from the first annotation based on the v1.0 assembly (Geissler‐Lee *et al*., 2009). The availability of the AspWood database (http://aspwood.popgenie.org/aspwood-v3.0/) and the aspen expression atlas (Sundell *et al*., 2015), as well as published aspen RNA‐Seq data (Immanen *et al*., 2016), provided evidence for the expression for 94% of the gene models, and the expression of 62% of the models in developing wood tissues, and enabled comparative analyses of expression. We used the CAZymes expression profiles during wood development and their relatedness to create corresponding co‐expression networks and to identify regulons composed of CAZymes and other genes. Transcriptional changes occurring during wood development provided several candidates for the biosynthesis of primary and secondary cell wall in *P. trichocarpa*. Our findings support the existence of two separate regulons for genes involved in secondary wall xylan biosynthesis: one strictly limited to secondary wall formation and another with a broader expression pattern encompassing the cambium and radial expansion zone. Moreover, the data provide support for the diversification of functions of the *Populus* secondary cellulose synthase paralogs *PtCesA8‐A* and *PtCesA8‐B*. The collection of tissue‐specific uncharacterized genes and transcription factors offers a rich source of targets for the future genetic improvement of woody plants.

## Experimental procedures

### Identification and annotation of CAZymes families in *P. trichocarpa* v3.0

The protein sequences from 41 335 loci of the *P. trichocarpa* genome (v3.0) were downloaded from the US Department of Energy (DOE) Joint Genome Institute (https://phytozome.jgi.doe.gov/pz/portal.html#!bulk?org=Org_Ptrichocarpa) and matched to over 1 million curated entries in the CAZy database as of November 2018 (http://www.cazy.org).

### Establishment of CAZyme expression matrix for comparative analyses

The high‐resolution RNA‐Seq data sets for the wild and glasshouse grown aspen (*Populus tremula* L.) are available from the PlantGenIE website (Sundell *et al*., 2015), and those for glasshouse‐grown hybrid aspen phloem, cambium and developing xylem are detailed by Immanen *et al*. (2016). The retrieved data were pre‐processed according to our guidelines as described by Delhomme *et al*. (http://www.epigenesys.eu/en/protocols/bio-informatics/1283-guidelines-for-rna-seq-data-analysis). Briefly, the quality of the raw sequence data was assessed using fastqc (http://www.bioinformatics.babraham.ac.uk/projects/fastqc/). Residual ribosomal RNA (rRNA) contamination was assessed and filtered using sortmerna 2.1 (with settings –log –fastx –sam –num_alignments 1 for both data sets, with the addition of –paired_in for the AspWood data set; Kopylova *et al*., 2012) using the rRNA sequences provided with sortmerna (rfam‐5s‐database‐id98.fasta, rfam‐5.8s‐database‐id98.fasta, silva‐arc‐16s‐database‐id95.fasta, silva‐bac‐16s‐database‐id85.fasta, silva‐euk‐18s‐database‐id95.fasta, silva‐arc‐23s‐database‐id98.fasta, silva‐bac‐23s‐database‐id98.fasta and silva‐euk‐28s‐database‐id98.fasta). Data were then filtered to remove adapters and trimmed for quality using trimmomatic 0.32 (with settings TruSeq3‐PE‐2.fa:2:30:10 LEADING:3 SLIDINGWINDOW:5:20 MINLEN:50; Bolger *et al*., 2014). After both filtering steps, fastqc was run again to ensure that no technical artefacts were introduced. Filtered reads were aligned to v3.0 of the *P. trichocarpa* genome (https://phytozome.jgi.doe.gov/pz/portal.html, November 2018), using star 2.5.2b (with non‐default settings –outSAMstrandField intronMotif –readFilesCommand zcat –outSAMmapqUnique 254 –quantMode TranscriptomeSAM –outFilterMultimapNmax100 –outReadsUnmapped Fastx –chimSegmentMin1 –outSAMtype BAM SortedByCoordinate –outWigType bedGraph –alignIntronMax11000; Dobin *et al*., 2013). The annotations obtained from the *P. trichocarpa* v3.0 GFF file were flattened to generate ‘synthetic’ gene models. This synthetic transcript GFF file and the star read alignments were used as input to the HTSeq (Anders *et al*., 2014) htseq‐count python utility to calculate exon‐based read count values. The htseq‐count utility takes only unique mapping reads into account. Because of their different characteristics (differences in sampling, sequencing instruments, etc.), the data sets were normalized independently. First, the data sets were normalized using variance stabilizing transformation (VST) in r 3.4.0 (R Core Team, 2015) using the bioconductor 3.4 (Gentleman *et al*., 2004) deseq2 package (v1.16.1; Love *et al*., 2014). Furthermore, the data from Immanen *et al*. (2016) were then filtered and samples corresponding to the zones of interest were identified (phloem, cambium and developing xylem) and selected from the three control trees: 4A, 4B and 6B. A likely artefact introduced by decreasing library sizes as samples moved inwards (towards the xylem) was identified and was corrected using a linear modeling approach. Finally, the data from both data sets were merged together using a sample‐based median‐centering approach. The resulting data were subjected to hierarchical clustering and the τ tissue/sample specificity score (Yanai *et al*., 2004) was calculated, both in r. The whole data analysis was performed using custom r scripts available from our GitHub repository (https://github.com/UPSCb/UPSCb/tree/master/manuscripts/Kumar2018) and the corresponding data are available from our FTP repository (ftp://ftp.plantgenie.org/Publications/Kumar2018).

To identify the poplar orthologs of characterized CAZyme genes of *A. thaliana*, the protein sequences were aligned using muscle (http://phylogeny.lirmm.fr/phylo_cgi/index.cgi) and phylogenic trees were constructed using the neighbor‐joining (NJ) method of mega 7 (Kumar *et al*., 2016) in default mode with a bootstrap test of 1000 replicates. *Arabidopsis thaliana* orthologs of the identified poplar genes were identified by blast (https://www.arabidopsis.org/Blast/).

### CAZymes involved in wood biosynthesis

The AspWood high‐spatial‐resolution RNA‐Seq data set was used for the functional analysis of CAZymes in wood formation (Sundell *et al*., 2017). The identity of wood developmental zones was based on the expression of the marker genes (Sundell *et al*., 2017). A heat map of CAZyme expression was constructed using the r 3.4.0 (R Core Team 2015) gplots package and the clustering was performed using the Ward.D2 method on Pearson distances. The custom scripts are available from the same GitHub repository described above. CAZyme gene clustering was kept from the published hierarchical clusters (Sundell *et al*., 2017). To evaluate the CAZymes transcriptome investment in the development of various tissues in wood, we calculated the average VST expression values for all samples covering specified developmental zones in tree 1.

### CAZymes networks

Co‐expression networks for all expressed CAZymes were obtained from the AspWood database (http://aspwood.popgenie.org/aspwood-v3.0/) and the corresponding Graphml file was obtained using the exnet tool (http://popgenie.org/exnet) at a Z‐score threshold of 5.0, and is available at ftp://ftp.plantgenie.org/Publications/Kumar2018. The co‐expression networks represented at AspWood were calculated using mutual information (MI) and context likelihood of relatedness (CLR), as explained in Sundell *et al*. (2017). The resulting Graphml file was further explored in cytoscape 3.4.0 (Shannon *et al*., 2003) for visualization, coloring and determining the first neighbors of functionally known ‘Guide Genes’ involved in the development of primary and secondary walls of the plant cell (Aoki *et al*., 2007).

### Association of secondary wall‐related CAZymes with other genes

Selected ‘Guide Genes’ were used to extract their corresponding networks containing first neighbors at threshold of 5.0 from AspWood. The similarity and differences in the neighborhoods of CAZymes were visualized using cytoscape 3.4.0.

### Data statement

This manuscript is based on publicly available data sets deposited at Phytozome (https://phytozome.jgi.doe.gov/pz/portal.html), CAZy (http://www.cazy.org) and AspWood (http://aspwood.popgenie.org/aspwood-v3.0/; Sundell *et al*., 2017). The CAZy annotation is provided in supplementary Tables S1–S8.

### Cell wall chemical analysis in different developmental zones of secondary xylem and phloem

Stem segments from the basal part of two glasshouse‐grown 6‐month‐old hybrid aspen trees (*P. tremula* L. × *tremuloides* Michx.), clone T89, were split into bark and wood core, and sequentially scraped on both exposed surfaces to obtain two sequential developmental stages of wood and secondary phloem differentiation. The rest of the wood core was analyzed as ‘mature wood’. The tissues were freeze‐dried, ground and analyzed for monosaccharide composition by alditol acetates (Englyst and Cummings, 1984) after hydrolysis in 2M TFA, for uronic acid content by a biphenyl assay (Filisetti‐Cozzi and Carpita, 1991), and for Klason lignin content as described previously (Gandla *et al*., 2015). The developmental trend was similar for the two trees analyzed, and results from one tree are shown in Figure 2 whereas the results for the other tree are shown in Figure S3.

## Funding information

We acknowledge VR and Formas grants to E.J.M., the SSF project ValueTree RBP14‐0011, the support from Vinnova (the Swedish Governmental Agency for Innovation Systems) and KAW (The Knut and Alice Wallenberg Foundation), and the Kempe post‐doc stipendium to V.K.

## Author contributions

VK performed most data analyses, prepared all figures and tables, and wrote the manuscript with EJM, MH and BH performed CAZyme re‐identification. ND, CM and NRS assisted with data retrieval and transcriptome analyses. P.I. was responsible for cell wall analyses. EJM conceived and coordinated the project, finalized the manuscript with contributions from all co‐authors, and agrees to serve as the author responsible for correspondence.

## Conflict of interest

The authors declare no conflicts of interest.

## Supporting information


**Figure S1.** Phylogenetic trees of selected CAZyme families in poplar and *A. thaliana*.

**Figure S2.** Variation of CAZymes expression across the wood‐forming zones of aspen.

**Figure S3.** Variability in cell wall composition across wood developmental zones for the second tree analyzed.

**Table S1.** List of annotated glycoside hydrolases (GHs) in *P. trichocarpa* genome v3.0.
**Table S2.** List of annotated glycosyl transferases (GTs) in *P. trichocarpa* genome v3.0.
**Table S3.** List of annotated polysaccharide lyases (PLs) in *P. trichocarpa* genome v3.0.
**Table S4.** List of annotated carbohydrate esterases (CEs) in *P. trichocarpa* genome v3.0.
**Table S5**. List of annotated auxiliary activities (AAs) in *P. trichocarpa* genome v3.0.
**Table S6.** List of annotated expansins (EXPN) in *P. trichocarpa* genome v3.0.
**Table S7.** List of annotated carbohydrate binding motifs (CBMs) in *P. trichocarpa* genome v3.0.
**Table S8.** *Arabidopsis thaliana* auxiliary activities (AAs).

**Table S9.** CAZyme expression matrix for comparative analyses.
**Table S10.** CAZymes expressed in developing secondary xylem and phloem of aspen.
**Table S11.** Number of CAZymes per family and per each expression cluster corresponding to wood developmental zones.
**Table S12.** List of CAZymes with documented and likely functions in cell wall biosynthesis and modification, and in starch and sugar metabolism.
**Table S13.** Number of CAZymes per each metabolic activities and per each expression cluster corresponding to wood developmental zones.
**Table S14.** List of genes from CAZymes‐based networks in wood‐forming aspen tissues.
**Table S15.** List of first neighbors of primary wall‐associated guide genes.
**Table S16.** List of first neighbors of secondary wall‐associated guide genes.
**Table S17.** List of first neighbors of secondary wall‐expressed selected CAZymes.
