# Supplementary material for: Poplar carbohydrate‐active enzymes: whole‐genome annotation and functional analyses based on RNA expression data
Source: Plant J. 2019 Jul 1;99(4):589–609. doi: 10.1111/tpj.14417 (PMC6852159; doi:10.1111/tpj.14417)
Supplement: Supplementary file 1 — Figure S1. Phylogenetic trees of selected CAZyme families in poplar and A. thaliana. [file TPJ-99-589-s001.pdf]

Figure S1. Phylogenetic trees of selected CAZyme families in poplar and *A. thaliana*. Protein sequences were aligned by MUSCLE (http://phylogeny.lirmm.fr/phylo_cgi/index.cgi) and phylogenic trees were constructed using Neighbor-Joining (NJ) method of MEGA7 in default mode with Bootstrap test of 1000 replicates. The numbers beside the branches correspond to % bootstrap values and are shown only for the main branches with at least 50 % support. The families are presented in alphabetical order.

AA1

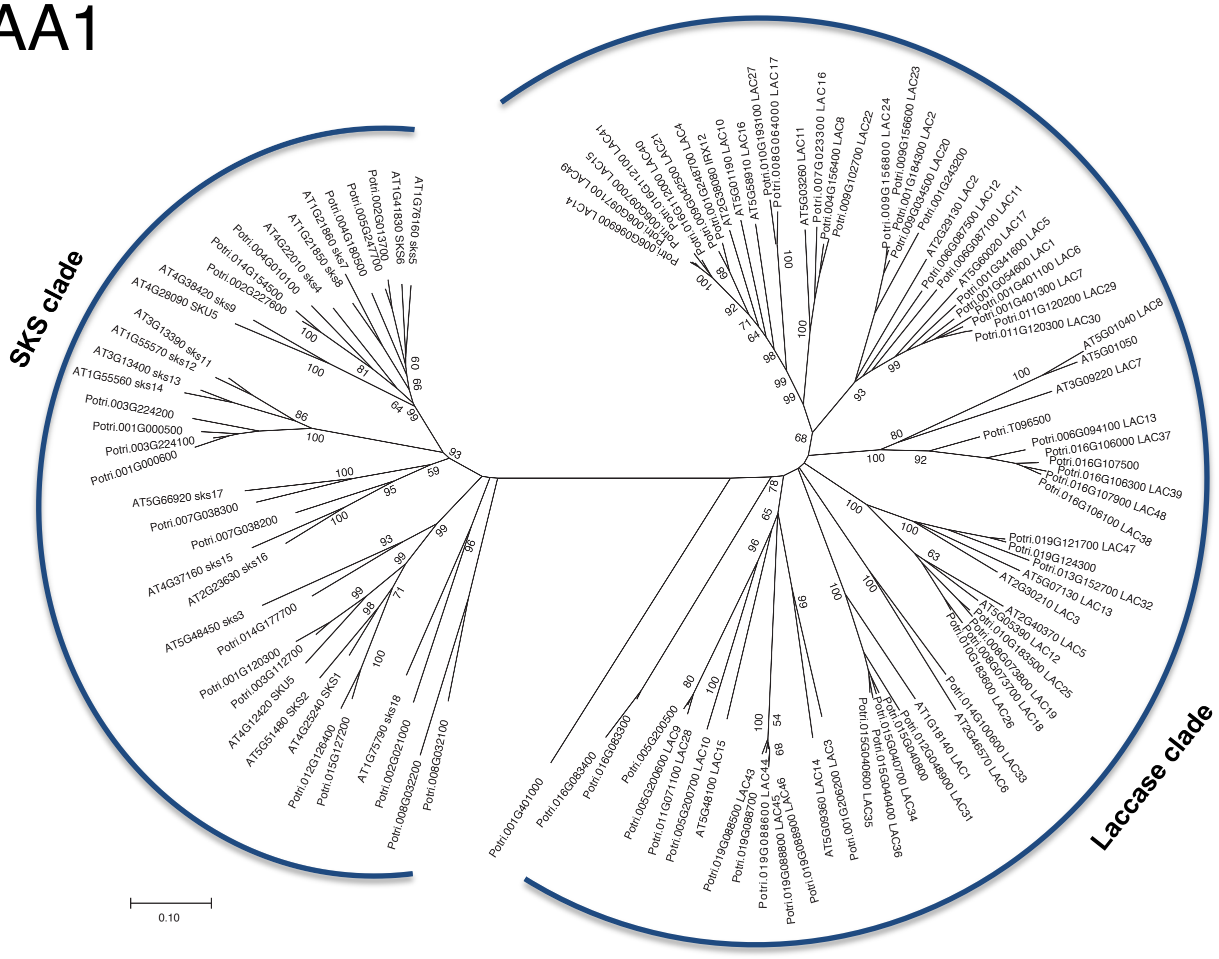

AA5

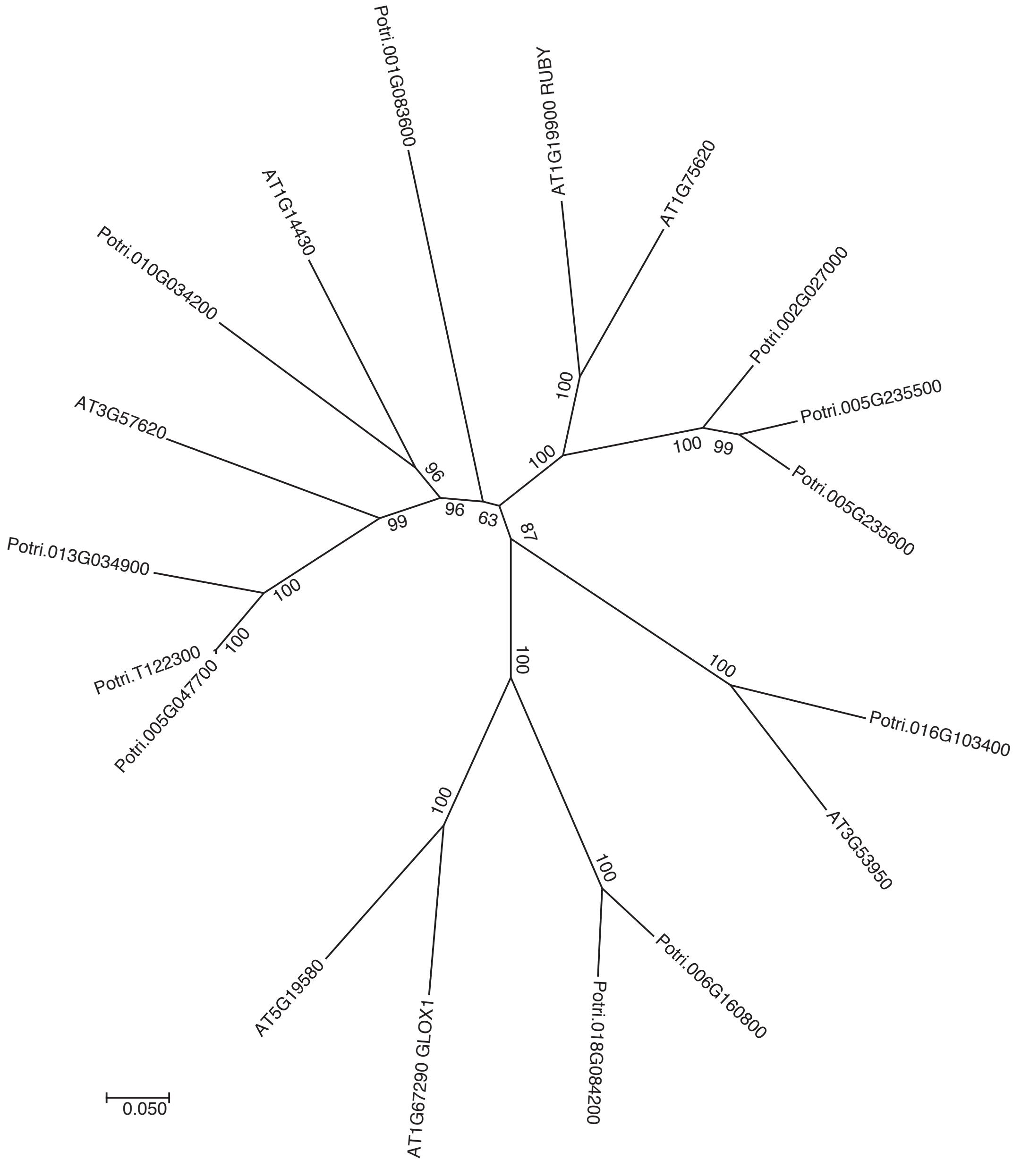

Potri.001G083600
AT1G19900 RUBY
AT1G75620
AT1G14430
Potri.010G034200
Potri.002G027000
Potri.005G235500
AT3G57620
Potri.005G235600
Potri.013G034900
Potri.T122300
Potri.005G047700
Potri.016G103400
AT3G53950
AT5G19580
AT1G67290 GLOX1
Potri.018G084200
Potri.006G160800
100
100
100
99
96
96
99
63
87
100
100
100
100
100
100
100
0.050

AA6

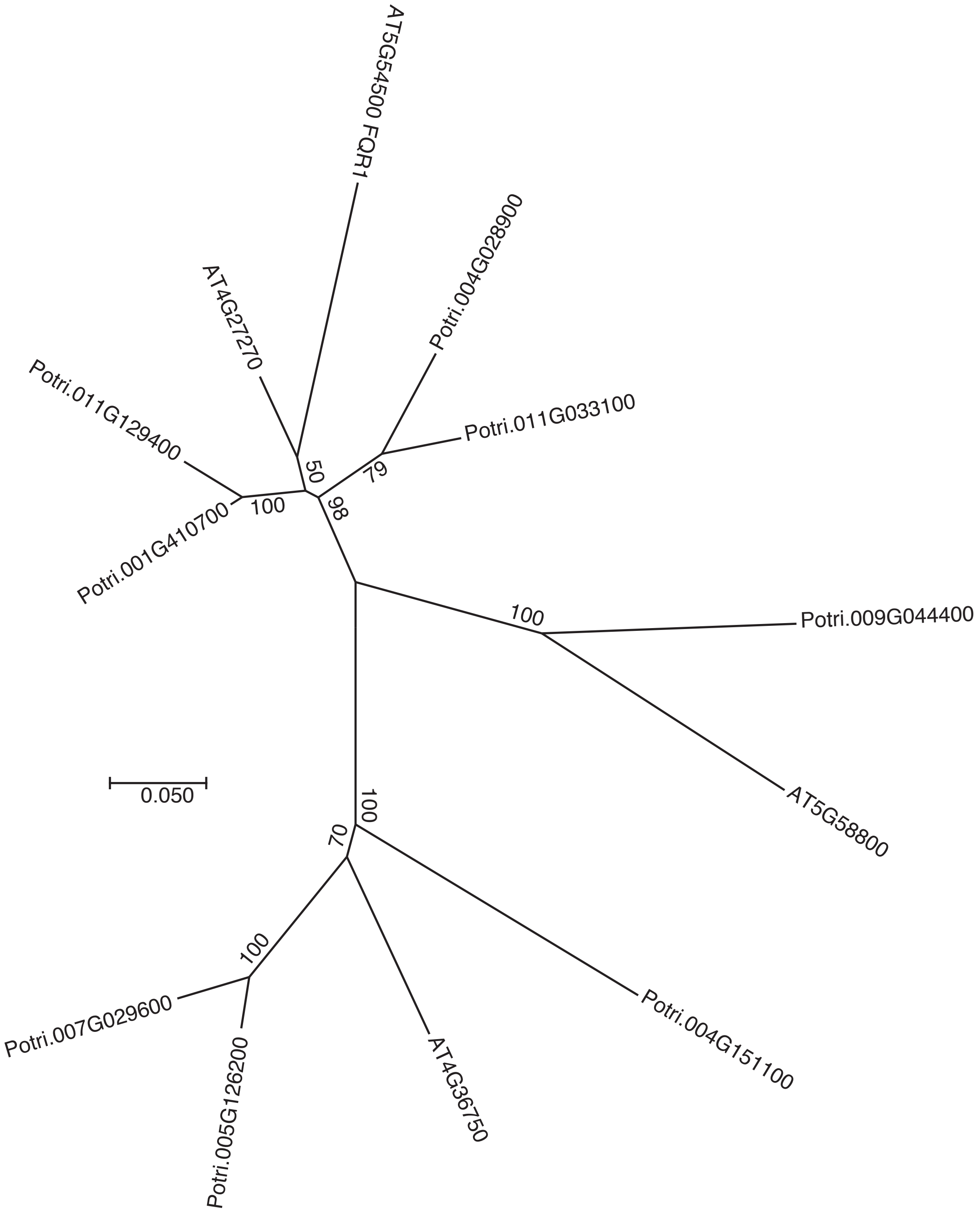
AT5G54500 FQR1
AT4G27270
Potri.004G028900
Potri.011G033100
Potri.011G129400
Potri.001G410700
50
79
98
100
100
Potri.009G044400
AT5G58800
0.050
100
70
100
Potri.007G029600
Potri.005G126200
AT4G36750
Potri.004G151100

AA7

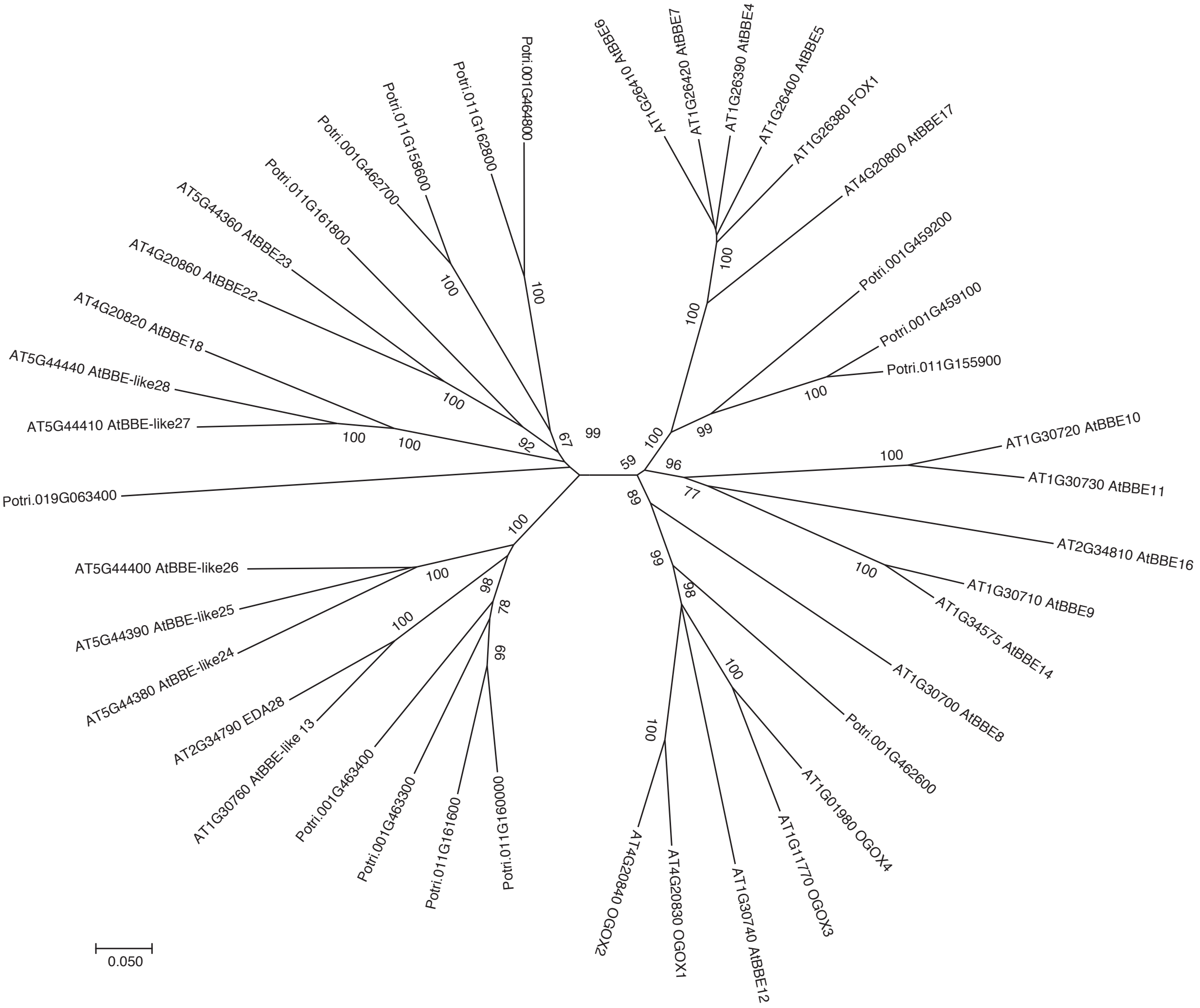

AT1G26410 AtBBE6
AT1G26420 AtBBE7
AT1G26390 AtBBE4
AT1G26400 AtBBE5
AT1G26380 FOX1
AT4G20800 AtBBE17
Potri.001G459200
Potri.001G459100
Potri.011G155900
AT1G30720 AtBBE10
AT1G30730 AtBBE11
AT2G34810 AtBBE16
AT1G30710 AtBBE9
AT1G34575 AtBBE14
AT1G30700 AtBBE8
Potri.001G462600
AT1G01980 OGOX4
AT1G11770 OGOX3
AT1G30740 AtBBE12
AT4G20830 OGOX1
AT4G20840 OGOX2
Potri.011G160000
Potri.011G161600
Potri.001G463300
Potri.001G463400
AT1G30760 AtBBE-like 13
AT2G34790 EDA28
AT5G44380 AtBBE-like24
AT5G44390 AtBBE-like25
AT5G44400 AtBBE-like26
Potri.019G063400
AT5G44410 AtBBE-like27
AT5G44440 AtBBE-like28
AT4G20820 AtBBE18
AT4G20860 AtBBE22
AT5G44360 AtBBE23
Potri.011G161800
Potri.001G462700
Potri.011G158600
Potri.011G162800
Potri.001G464800
0.050

GH3

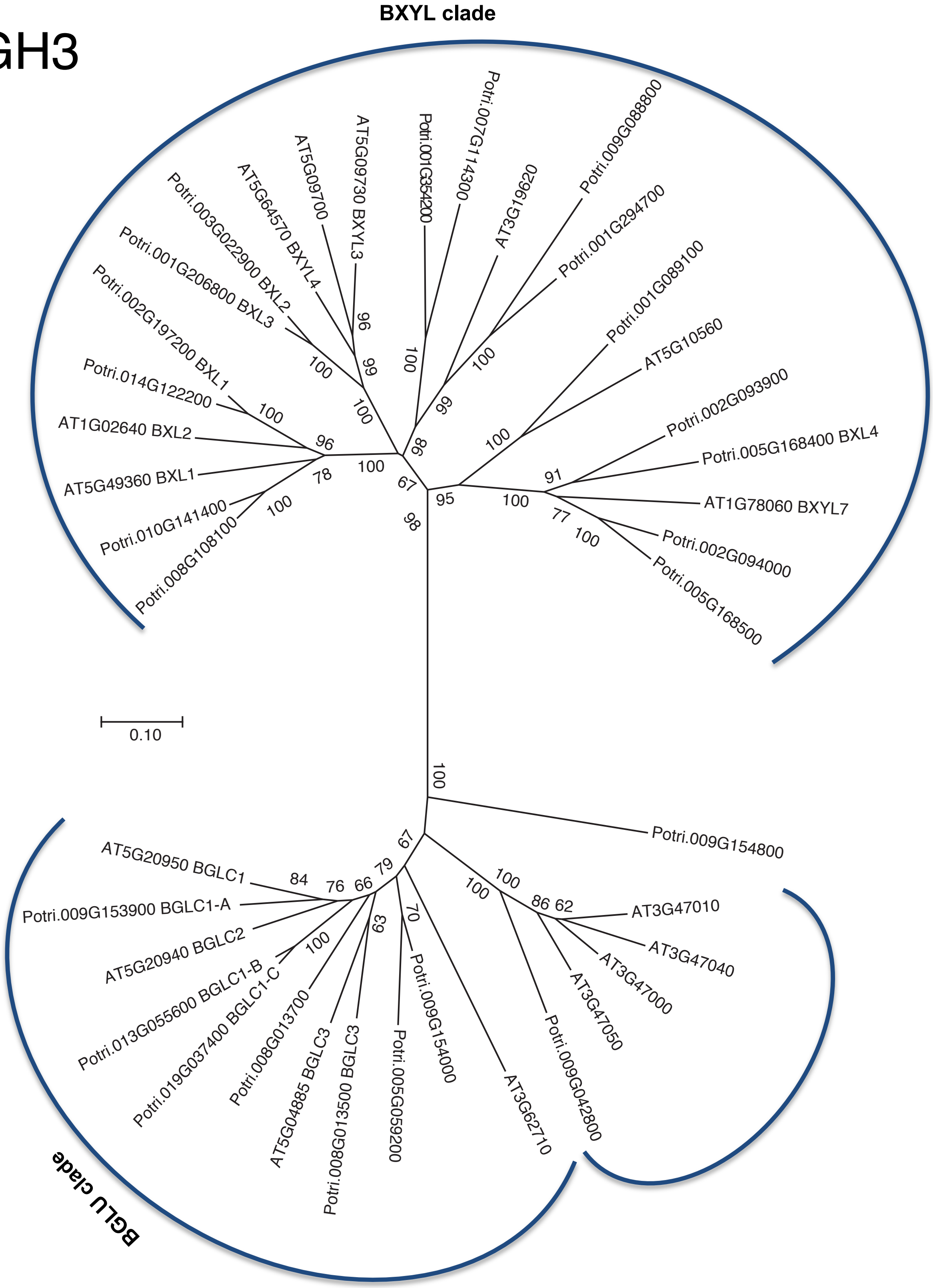

BXYL clade
Potri.007G114300
Potri.009G088800
AT5G09730 BXYL3
AT5G09700
AT3G19620
Potri.001G294700
AT5G64570 BXYL4
Potri.003G022900 BXL2
Potri.001G089100
Potri.001G206800 BXL3
AT5G10560
Potri.002G197200 BXL1
Potri.014G122200
Potri.002G093900
AT1G02640 BXL2
Potri.005G168400 BXL4
AT5G49360 BXL1
AT1G78060 BXYL7
Potri.010G141400
Potri.002G094000
Potri.008G108100
Potri.005G168500
0.10
Potri.009G154800
AT5G20950 BGLC1
Potri.009G153900 BGLC1-A
AT3G47010
AT5G20940 BGLC2
AT3G47040
Potri.013G055600 BGLC1-B
AT3G47000
Potri.019G037400 BGLC1-C
AT3G47050
Potri.008G013700
Potri.009G154000
AT5G04885 BGLC3
Potri.008G013500 BGLC3
Potri.005G059200
AT3G62710
Potri.009G042800
BGLU clade

GH13

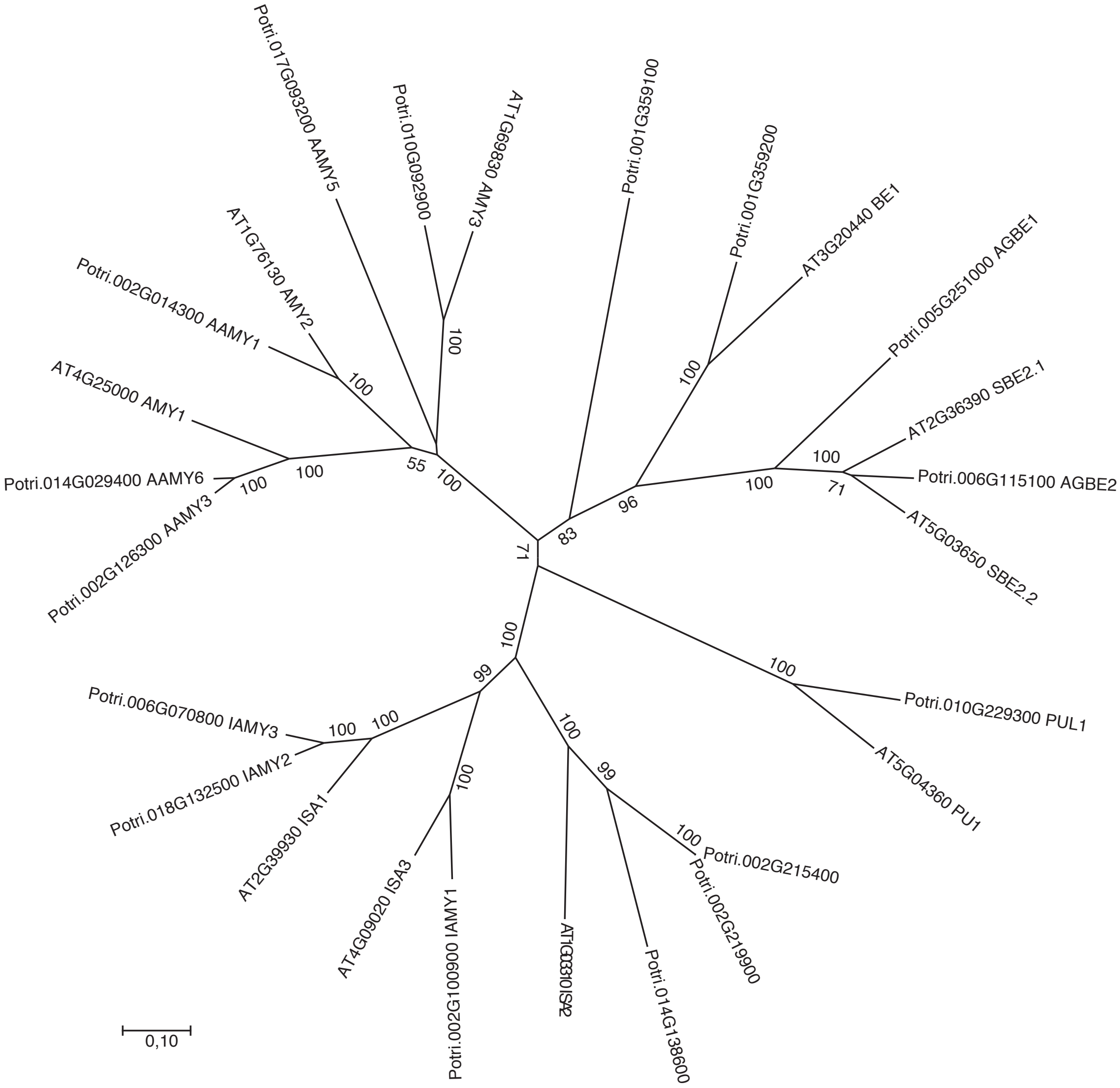

Potri.017G093200 AAMY5
Potri.010G092900
AT1G69830 AMY3
Potri.001G359100
Potri.001G359200
AT3G20440 BE1
Potri.005G251000 AGBE1
AT1G76130 AMY2
Potri.002G014300 AAMY1
AT4G25000 AMY1
Potri.014G029400 AAMY6
Potri.002G126300 AAMY3
AT2G36390 SBE2.1
Potri.006G115100 AGBE2
AT5G03650 SBE2.2
Potri.010G229300 PUL1
AT5G04360 PU1
Potri.006G070800 IAMY3
Potri.018G132500 IAMY2
AT2G39930 ISA1
AT4G09020 ISA3
Potri.002G100900 IAMY1
Potri.014G138600
Potri.002G215400
Potri.002G219900
100
55
100
100
100
100
100
100
96
83
100
100
71
71
100
100
99
100
100
100
100
99
100
100
0,10

GH14

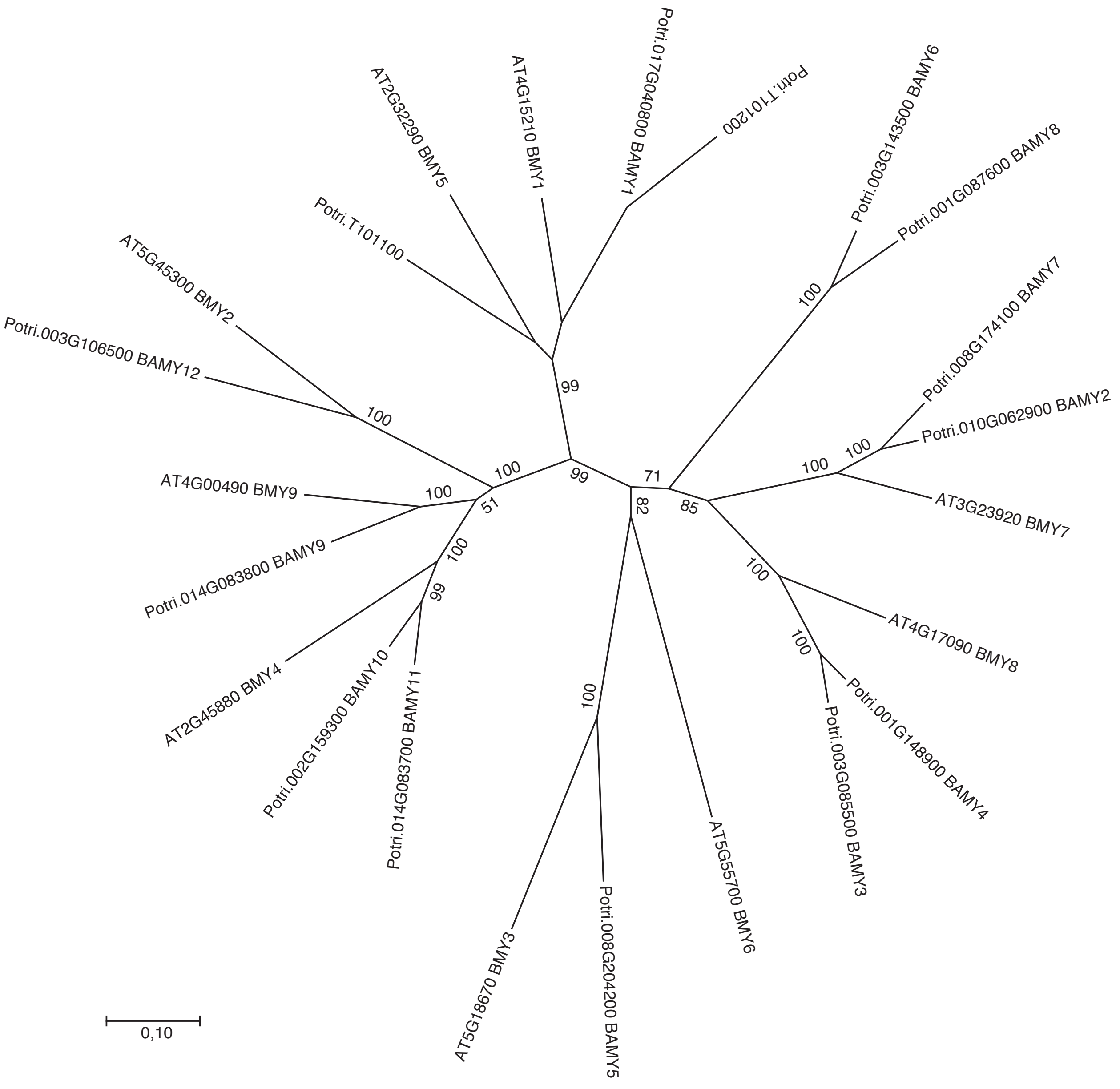

AT2G32290 BMY5
AT4G15210 BMY1
Potri.017G040800 BAMY1
Potri.T101200
Potri.003G143500 BAMY6
Potri.001G087600 BAMY8
Potri.T101100
AT5G45300 BMY2
Potri.003G106500 BAMY12
Potri.008G174100 BAMY7
Potri.010G062900 BAMY2
AT3G23920 BMY7
AT4G00490 BMY9
Potri.014G083800 BAMY9
AT2G45880 BMY4
Potri.002G159300 BAMY10
Potri.014G083700 BAMY11
AT4G17090 BMY8
Potri.001G148900 BAMY4
Potri.003G085500 BAMY3
AT5G55700 BMY6
Potri.008G204200 BAMY5
AT5G18670 BMY3
0,10

GH31

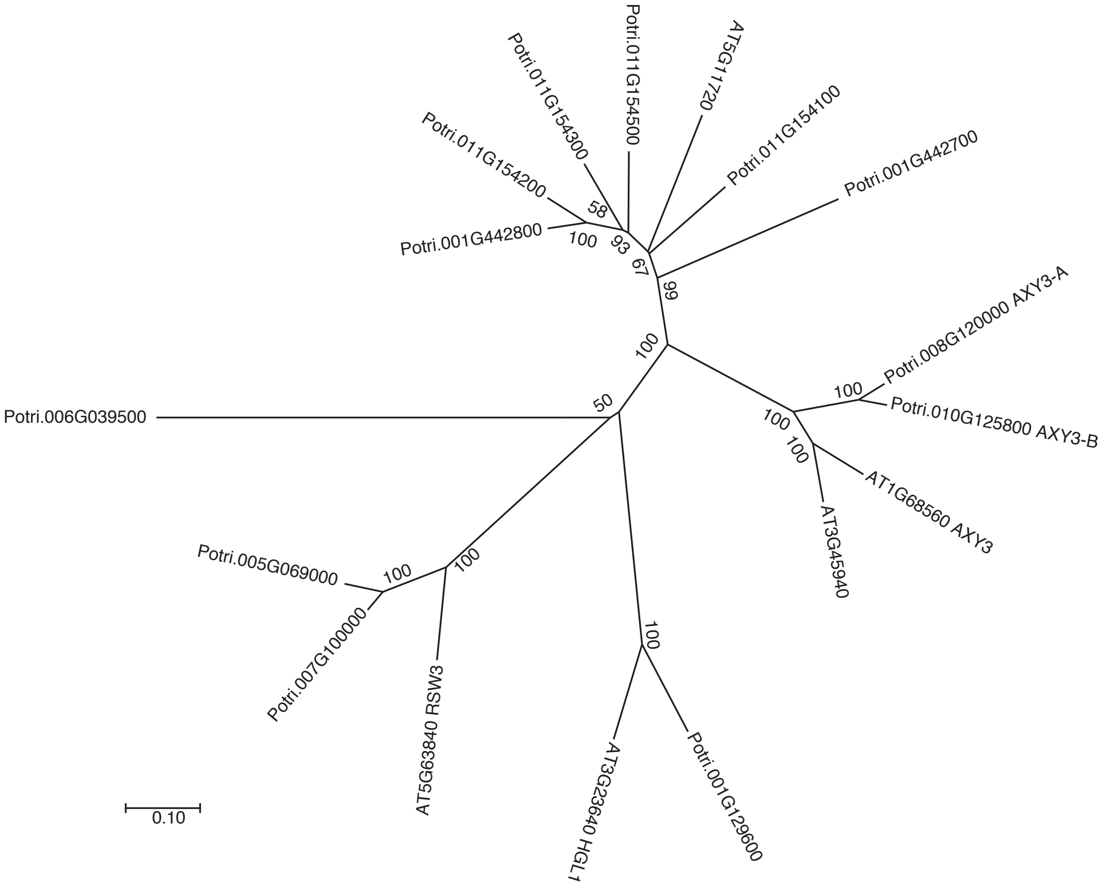
Potri.011G154300
Potri.011G154500
AT5G11720
Potri.011G154100
Potri.001G442700
Potri.011G154200
Potri.001G442800
58
100
93
67
99
100
Potri.008G120000 AXY3-A
Potri.010G125800 AXY3-B
100
100
100
AT1G68560 AXY3
AT3G45940
Potri.006G039500
50
Potri.005G069000
100
100
Potri.007G100000
AT5G63840 RSW3
100
AT3G23640 HGL1
Potri.001G129600
0.10

GH32

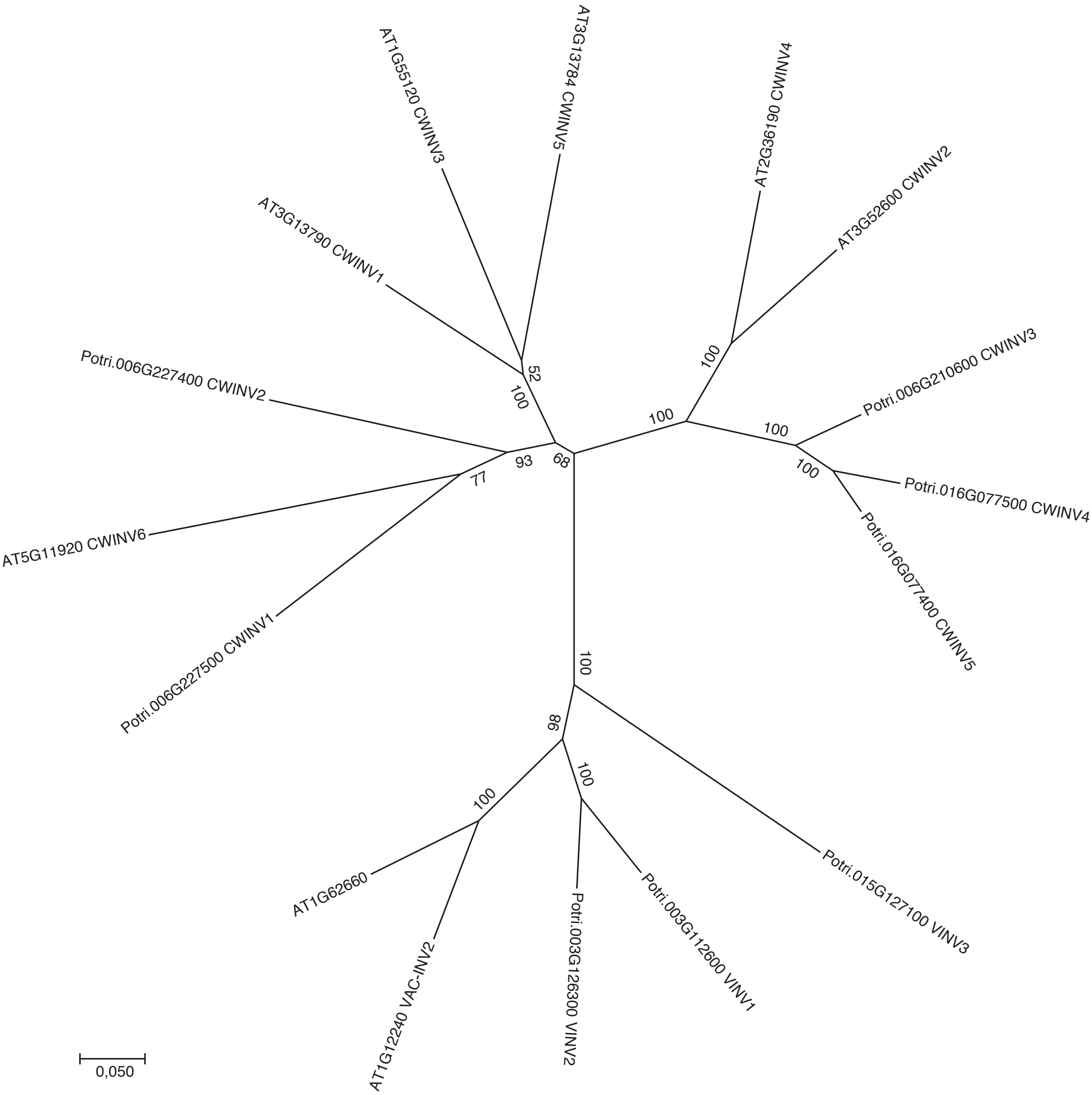

AT1G55120 CWINV3
AT3G13784 CWINV5
AT2G36190 CWINV4
AT3G52600 CWINV2
AT3G13790 CWINV1
Potri.006G227400 CWINV2
Potri.006G210600 CWINV3
Potri.016G077500 CWINV4
Potri.016G077400 CWINV5
AT5G11920 CWINV6
Potri.006G227500 CWINV1
Potri.015G127100 VINV3
Potri.003G112600 VINV1
Potri.003G126300 VINV2
AT1G62660
AT1G12240 VAC-INV2
52
100
100
100
100
100
93
68
77
100
98
100
100
0,050

GH35

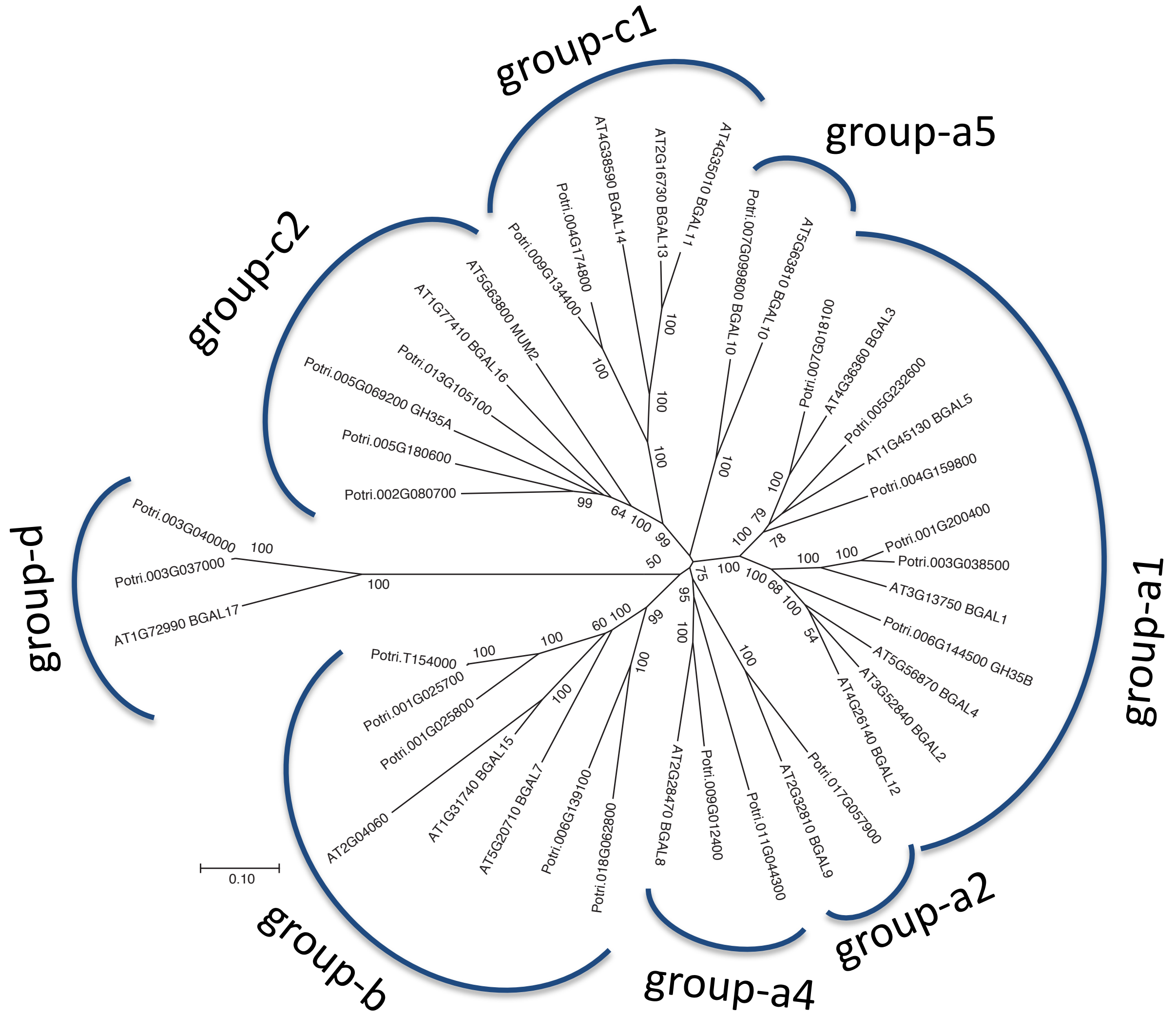

group-c1
group-a5
group-c2
group-d
group-a1
group-b
group-a4
group-a2
AT4G38590 BGAL14
AT2G16730 BGAL13
AT4G35010 BGAL11
Potri.004G174800
Potri.009G134400
AT5G63800 MUM2
AT1G77410 BGAL16
Potri.013G105100
Potri.005G069200 GH35A
Potri.005G180600
Potri.002G080700
Potri.007G099800 BGAL10
AT5G63810 BGAL10
Potri.007G018100
AT4G36360 BGAL3
Potri.005G232600
AT1G45130 BGAL5
Potri.004G159800
Potri.001G200400
Potri.003G038500
AT3G13750 BGAL1
Potri.006G144500 GH35B
AT5G56870 BGAL4
AT3G52840 BGAL2
AT4G26140 BGAL12
Potri.017G057900
AT2G32810 BGAL9
Potri.011G044300
Potri.009G012400
AT2G28470 BGAL8
Potri.018G062800
Potri.006G139100
AT5G20710 BGAL7
AT1G31740 BGAL15
AT2G04060
Potri.001G025800
Potri.001G025700
Potri.T154000
Potri.003G040000
Potri.003G037000
AT1G72990 BGAL17
0.10

GH77

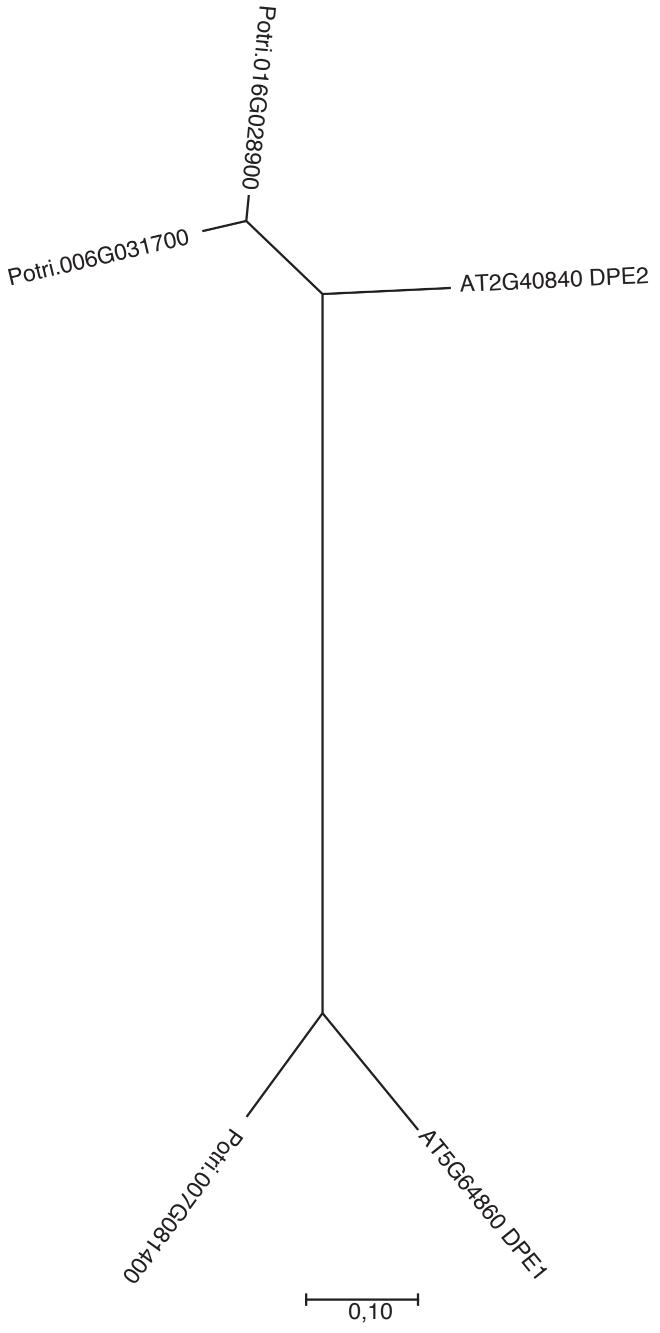

Potri.016G028900
Potri.006G031700
AT2G40840 DPE2
Potri.007G081400
AT5G64860 DPE1
0,10

GH79

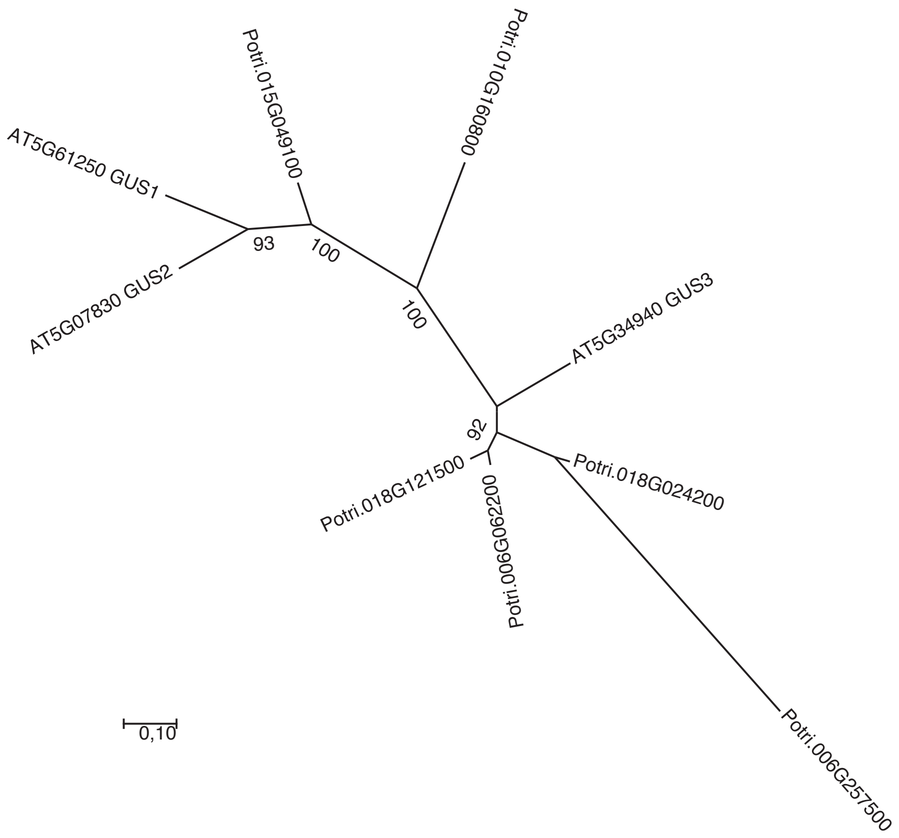
Potri.015G049100
Potri.010G160800
AT5G61250 GUS1
AT5G07830 GUS2
93
100
100
AT5G34940 GUS3
92
Potri.018G121500
Potri.006G062200
Potri.018G024200
Potri.006G257500
0,10

GH95

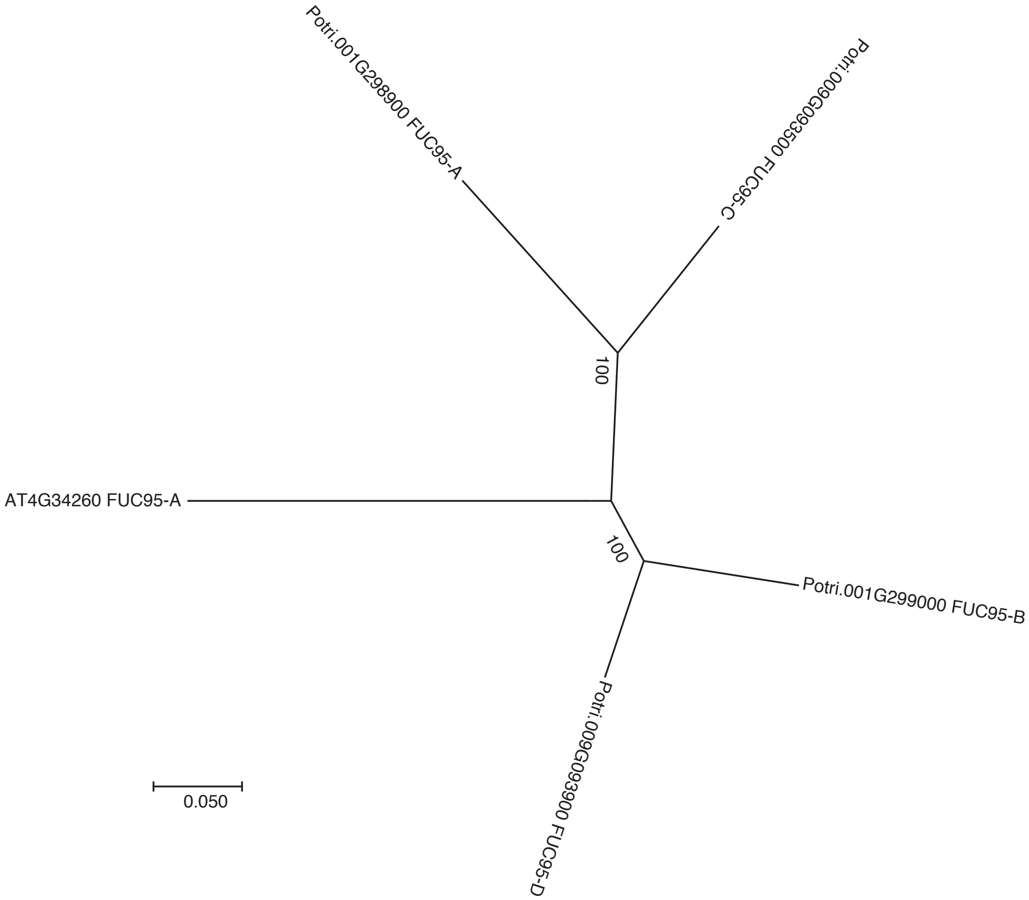
Potri.001G298900 FUC95-A
Potri.009G093500 FUC95-C
100
AT4G34260 FUC95-A
100
Potri.001G299000 FUC95-B
Potri.009G093900 FUC95-D
0.050

GH100

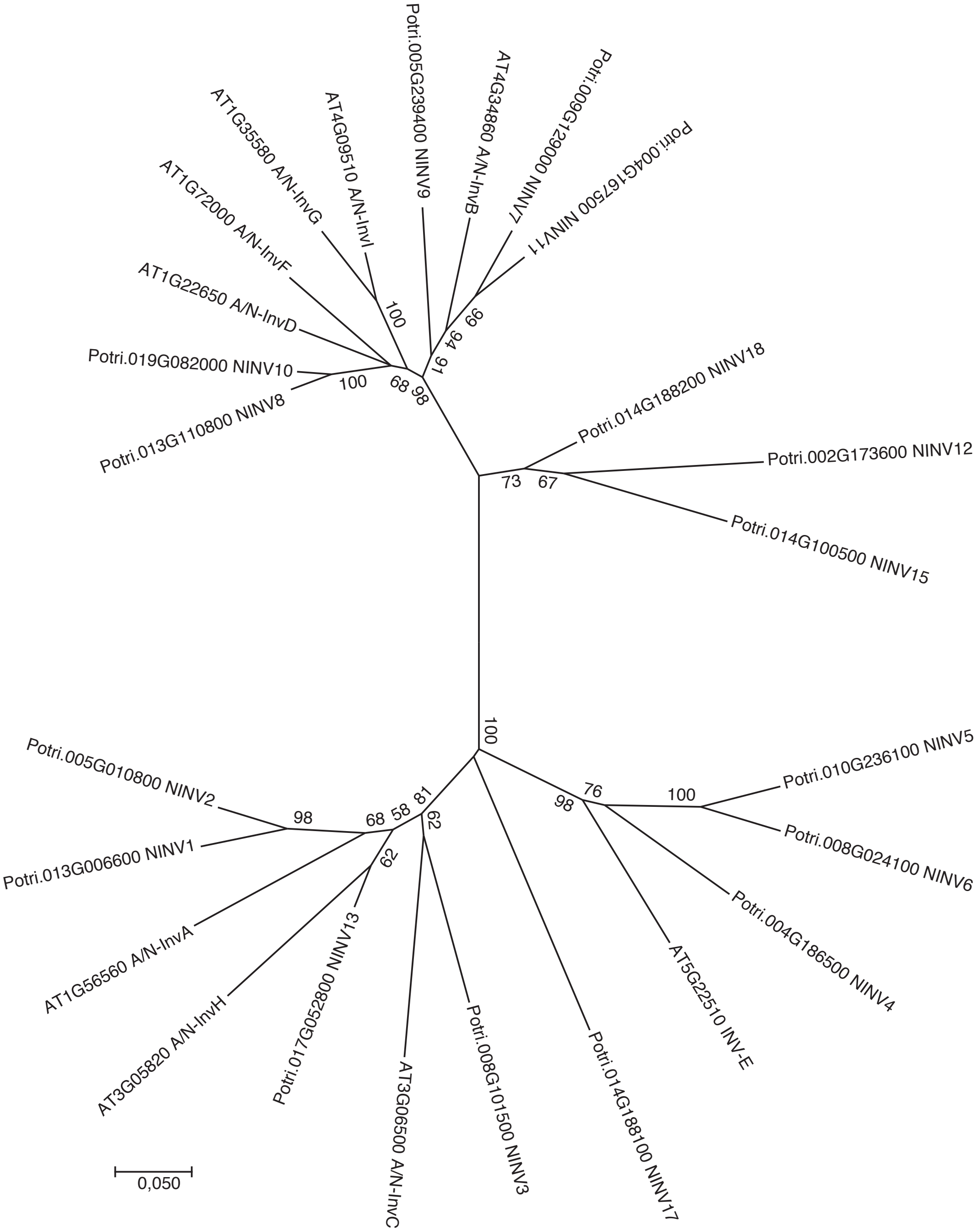

Potri.005G239400 NINV9
AT4G34860 A/N-InvB
Potri.009G129000 NINV7
Potri.004G167500 NINV11
AT4G09510 A/N-InvI
AT1G35580 A/N-InvG
AT1G72000 A/N-InvF
AT1G22650 A/N-InvD
Potri.019G082000 NINV10
Potri.013G110800 NINV8
Potri.014G188200 NINV18
Potri.002G173600 NINV12
Potri.014G100500 NINV15
Potri.005G010800 NINV2
Potri.013G006600 NINV1
AT1G56560 A/N-InvA
AT3G05820 A/N-InvH
Potri.017G052800 NINV13
AT3G06500 A/N-InvC
Potri.008G101500 NINV3
Potri.014G188100 NINV17
AT5G22510 INV-E
Potri.004G186500 NINV4
Potri.010G236100 NINV5
Potri.008G024100 NINV6
0,050

GH146

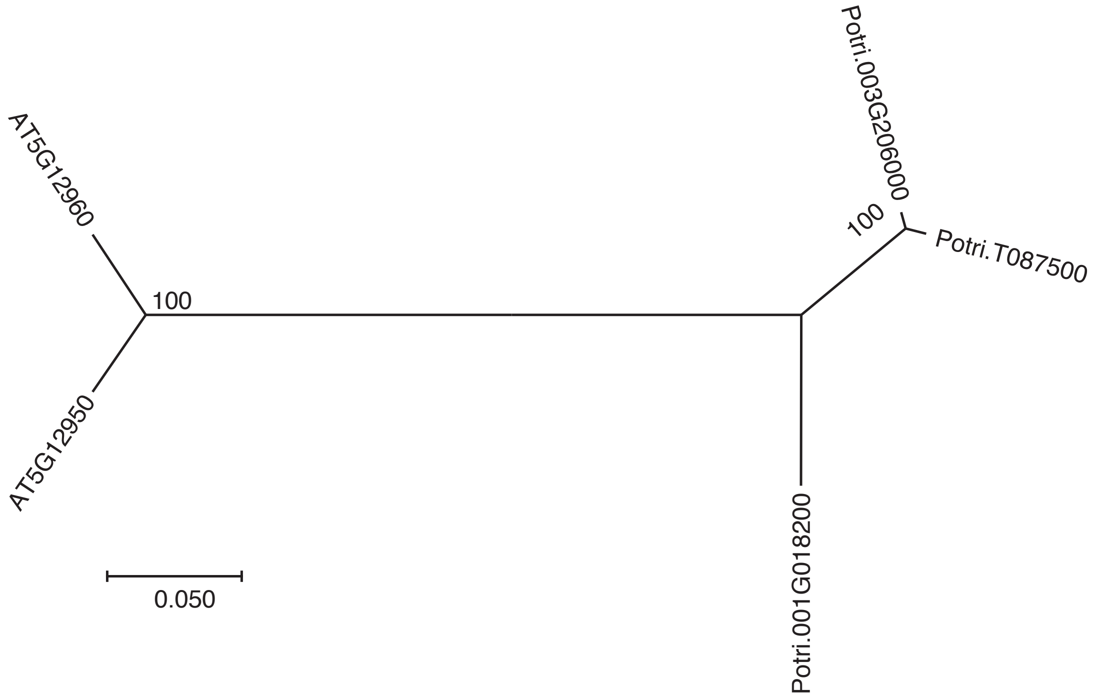

AT5G12960
AT5G12950
100
100
Potri.003G206000
Potri.T087500
Potri.001G018200
0.050

GH152

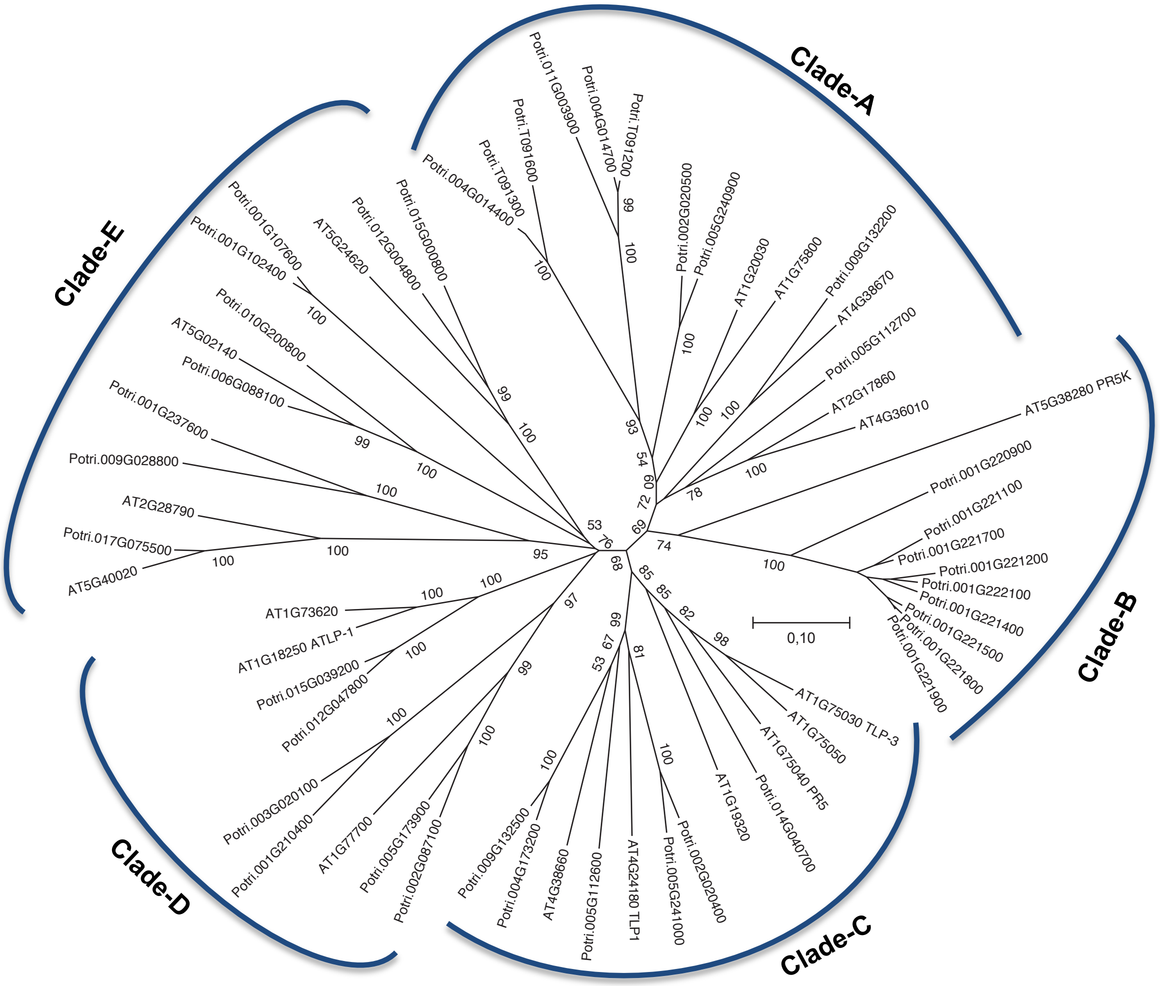

Clade-A
Clade-B
Clade-C
Clade-D
Clade-E
0,10
Potri.T091200
Potri.004G014700
Potri.011G003900
Potri.T091600
Potri.T091300
Potri.004G014400
Potri.002G020500
Potri.005G240900
AT1G20030
AT1G75800
Potri.009G132200
AT4G38670
Potri.005G112700
AT2G17860
AT4G36010
AT5G38280 PR5K
Potri.001G220900
Potri.001G221100
Potri.001G221700
Potri.001G221200
Potri.001G222100
Potri.001G221400
Potri.001G221500
Potri.001G221800
Potri.001G221900
AT1G75030 TLP-3
AT1G75050
AT1G75040 PR5
Potri.014G040700
AT1G19320
Potri.002G020400
Potri.005G241000
AT4G24180 TLP1
Potri.005G112600
AT4G38660
Potri.004G173200
Potri.009G132500
Potri.002G087100
Potri.005G173900
AT1G77700
Potri.001G210400
Potri.003G020100
Potri.012G047800
Potri.015G039200
AT1G18250 ATLP-1
AT1G73620
AT5G40020
Potri.017G075500
AT2G28790
Potri.009G028800
Potri.001G237600
Potri.006G088100
AT5G02140
Potri.010G200800
Potri.001G102400
Potri.001G107600
AT5G24620
Potri.012G004800
Potri.015G000800

GT2

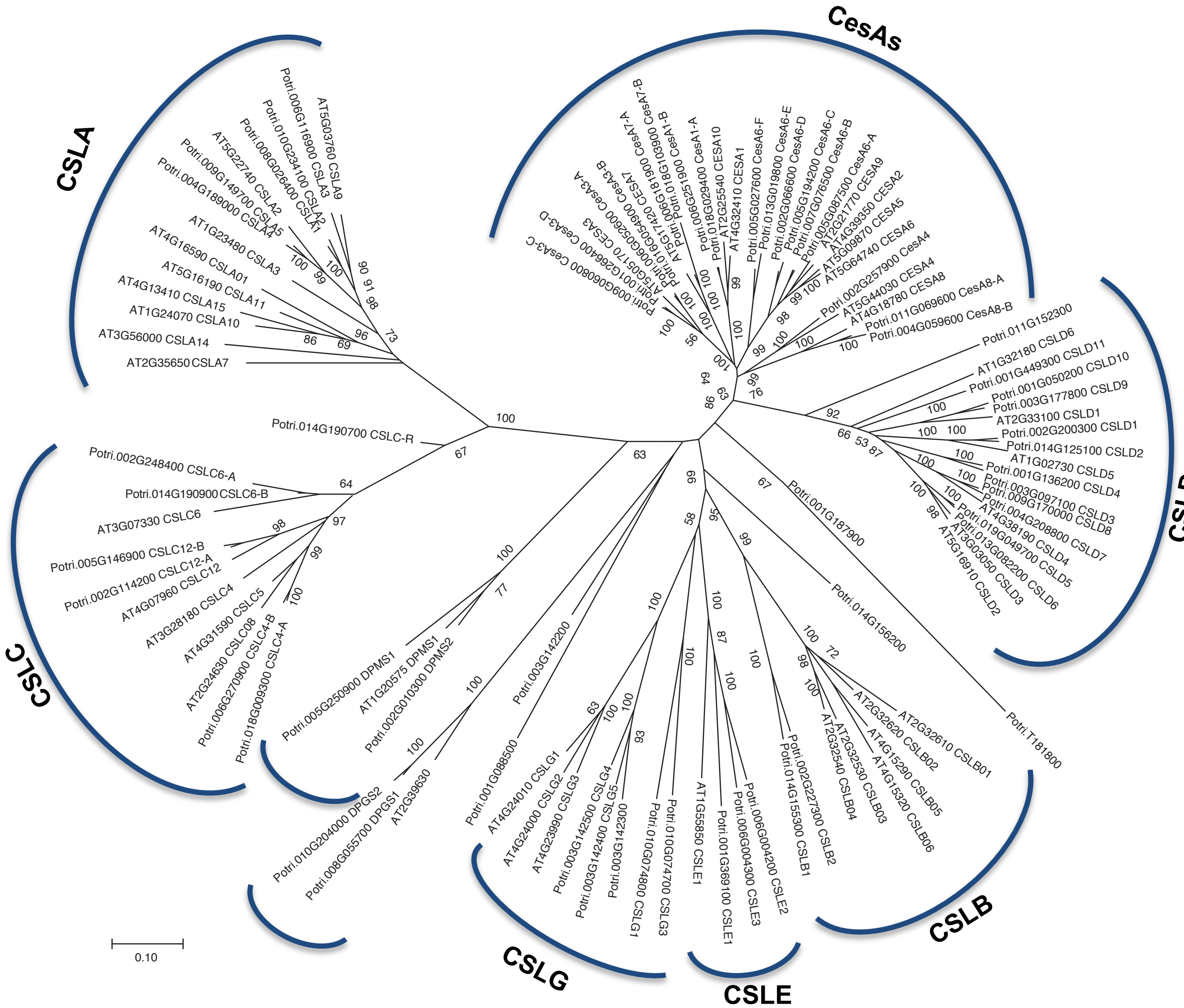

GT4

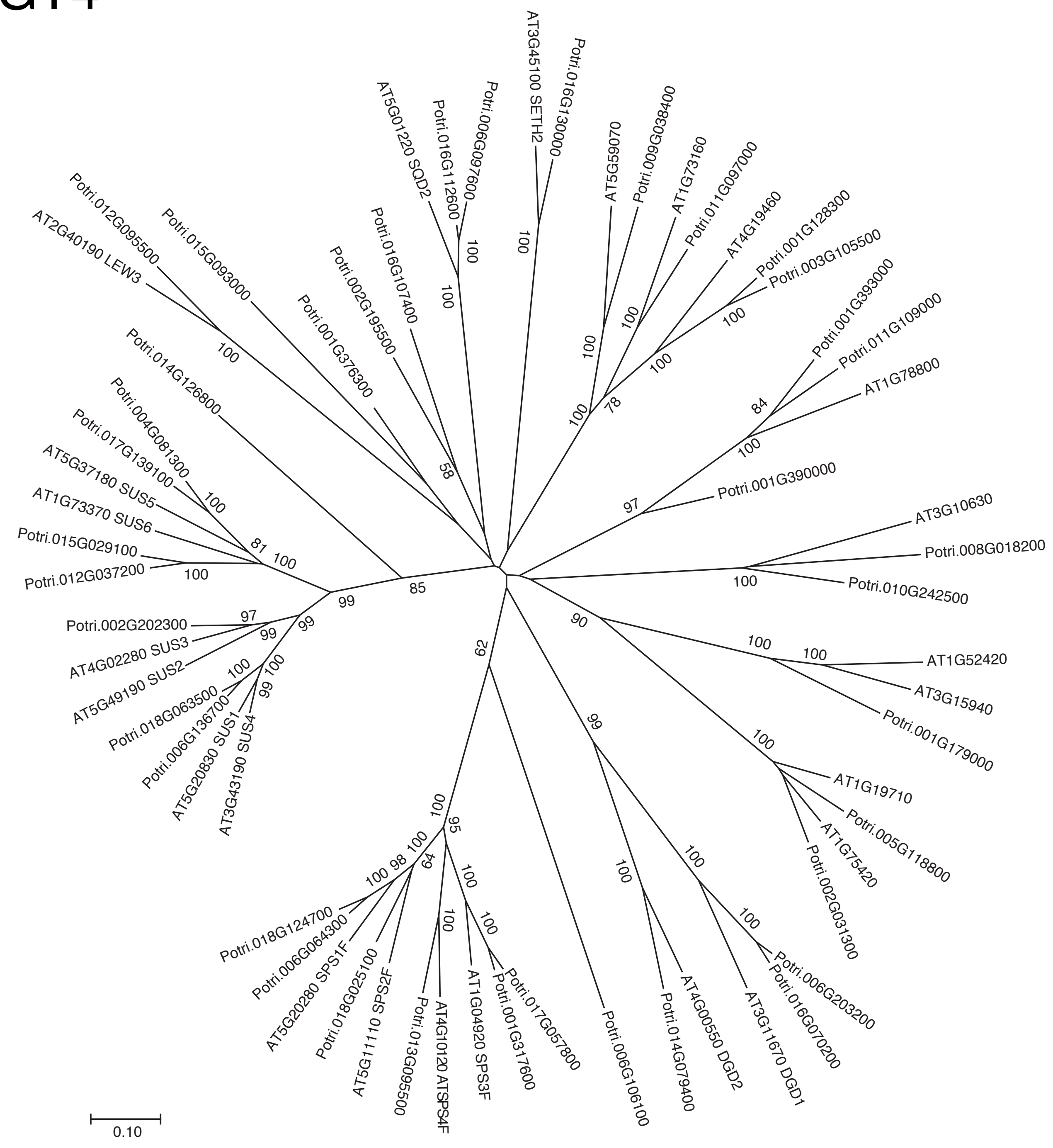

AT3G45100 SETH2
Potri.016G130000
Potri.006G097600
Potri.016G112600
AT5G01220 SQD2
AT5G59070
Potri.009G038400
AT1G73160
Potri.011G097000
AT4G19460
Potri.001G128300
Potri.003G105500
Potri.001G393000
Potri.011G109000
AT1G78800
Potri.001G390000
AT3G10630
Potri.008G018200
Potri.010G242500
AT1G52420
AT3G15940
Potri.001G179000
AT1G19710
Potri.005G118800
AT1G75420
Potri.002G031300
Potri.006G203200
Potri.016G070200
AT3G11670 DGD1
AT4G00550 DGD2
Potri.014G079400
Potri.006G106100
Potri.017G057800
Potri.001G317600
AT1G04920 SPS3F
AT4G10120 ATSPS4F
Potri.013G095500
AT5G11110 SPS2F
Potri.018G025100
AT5G20280 SPS1F
Potri.006G064300
Potri.018G124700
AT3G43190 SUS4
AT5G20830 SUS1
Potri.006G136700
Potri.018G063500
AT5G49190 SUS2
AT4G02280 SUS3
Potri.002G202300
Potri.012G037200
Potri.015G029100
AT1G73370 SUS6
AT5G37180 SUS5
Potri.017G139100
Potri.004G081300
Potri.014G126800
Potri.001G376300
Potri.002G195500
Potri.016G107400
Potri.015G093000
Potri.012G095500
AT2G40190 LEW3
0.10

GT5

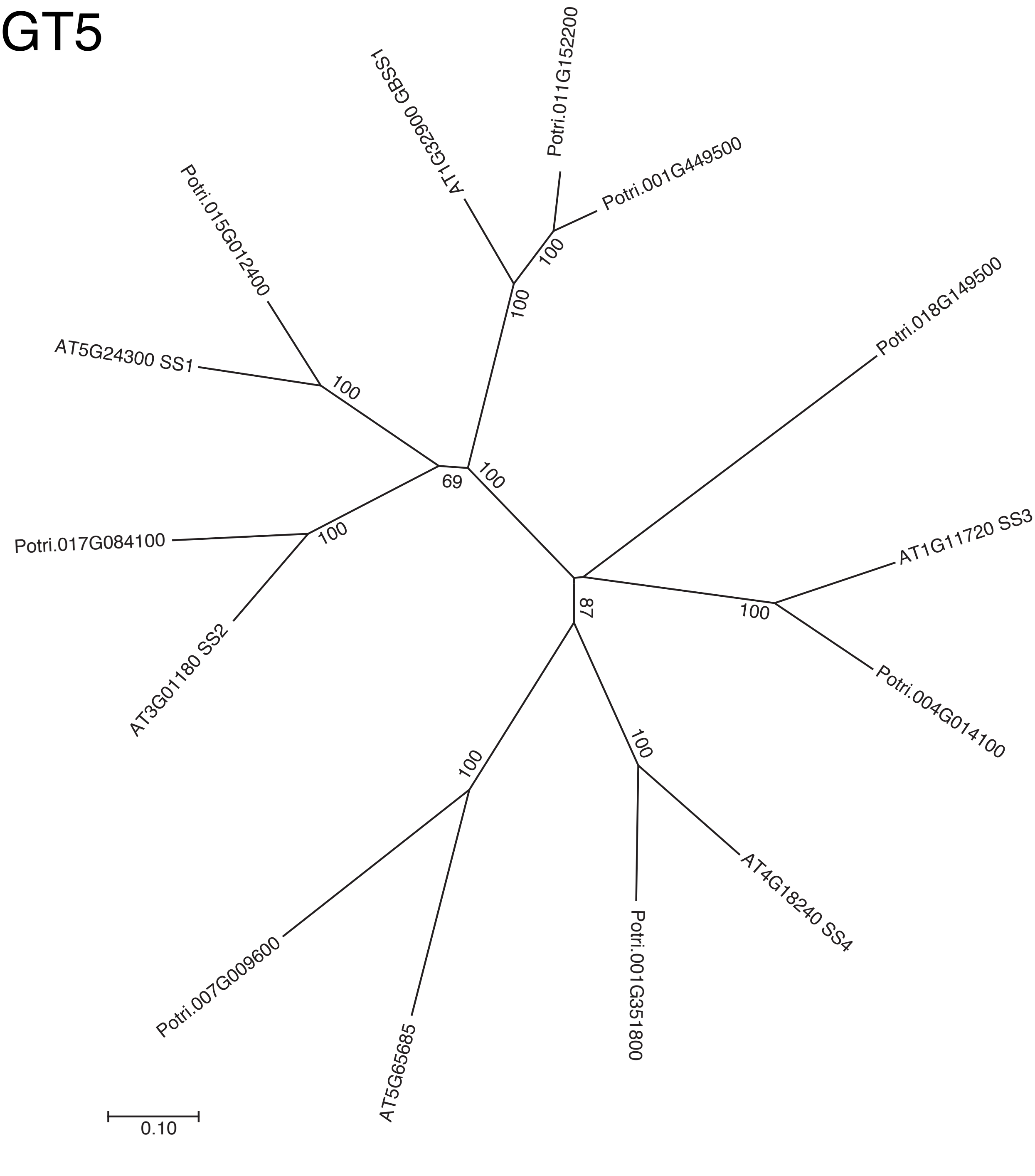

AT1G32900 GBSS1
Potri.011G152200
Potri.001G449500
Potri.015G012400
AT5G24300 SS1
Potri.018G149500
Potri.017G084100
AT3G01180 SS2
AT1G11720 SS3
Potri.004G014100
AT4G18240 SS4
Potri.001G351800
Potri.007G009600
AT5G65685
100
100
100
100
69
100
87
100
100
100
0.10

GT8

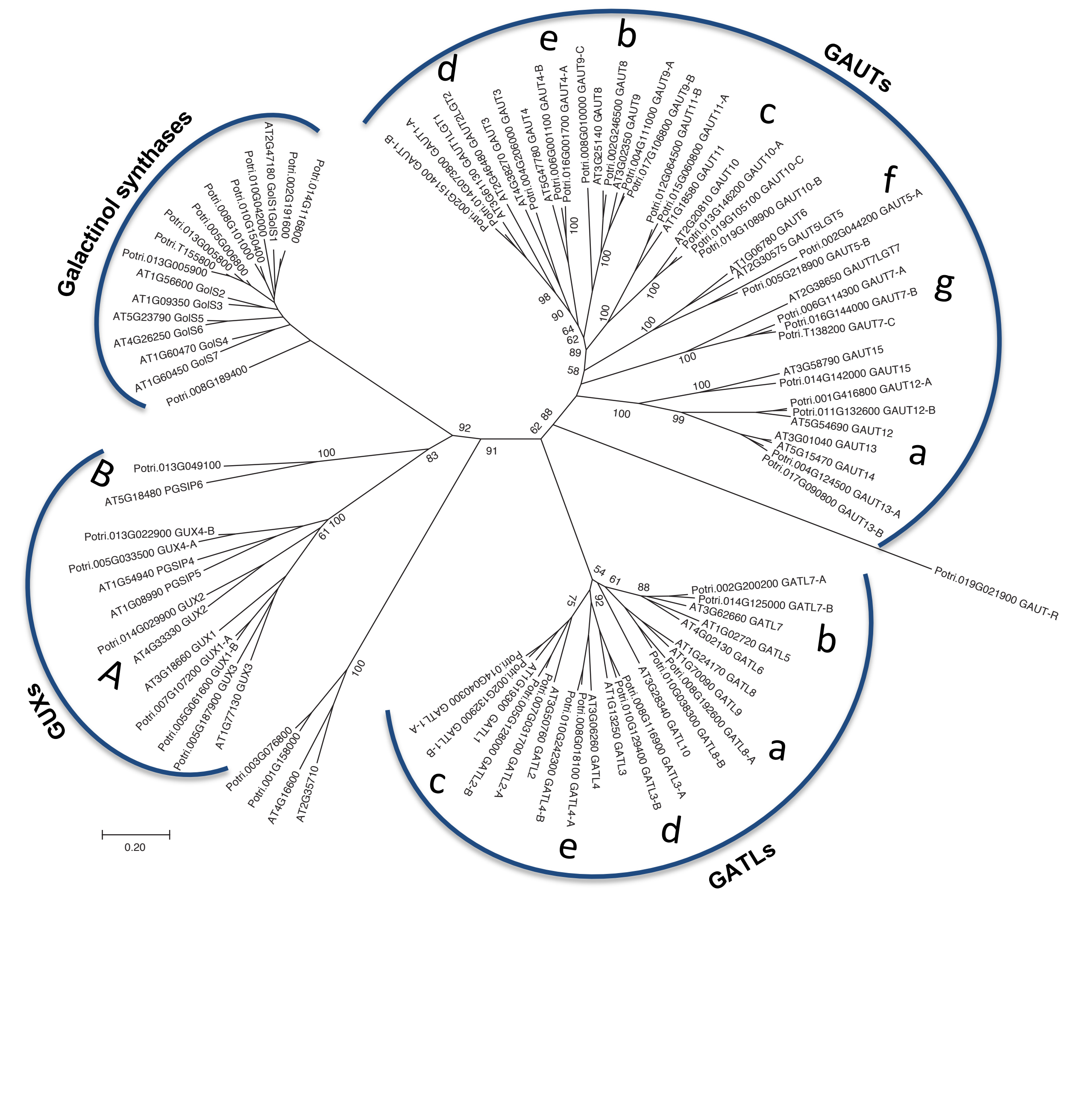

Galactinol synthases
GAUTs
GATLs
GUXs
Potri.019G021900 GAUT-R
Potri.013G049100
AT5G18480 PGSIP6
Potri.008G189400
0.20

GT20

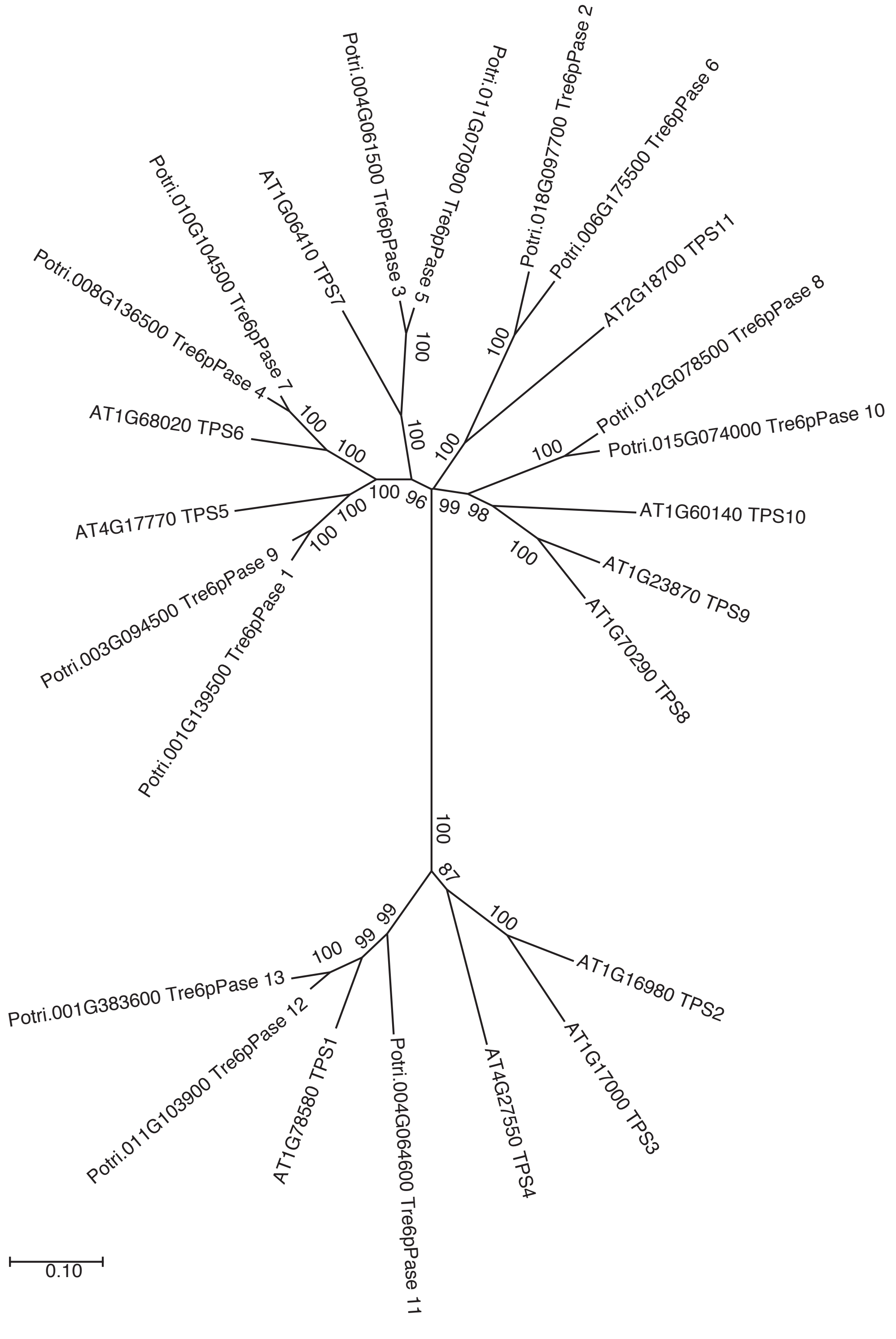
Potri.004G061500 Tre6pPase 3
Potri.011G070900 Tre6pPase 5
Potri.018G097700 Tre6pPase 2
Potri.006G175500 Tre6pPase 6
AT2G18700 TPS11
Potri.012G078500 Tre6pPase 8
Potri.015G074000 Tre6pPase 10
AT1G60140 TPS10
AT1G23870 TPS9
AT1G70290 TPS8
AT1G06410 TPS7
Potri.010G104500 Tre6pPase 7
Potri.008G136500 Tre6pPase 4
AT1G68020 TPS6
AT4G17770 TPS5
Potri.003G094500 Tre6pPase 9
Potri.001G139500 Tre6pPase 1
Potri.001G383600 Tre6pPase 13
Potri.011G103900 Tre6pPase 12
AT1G78580 TPS1
Potri.004G064600 Tre6pPase 11
AT4G27550 TPS4
AT1G17000 TPS3
AT1G16980 TPS2
0.10

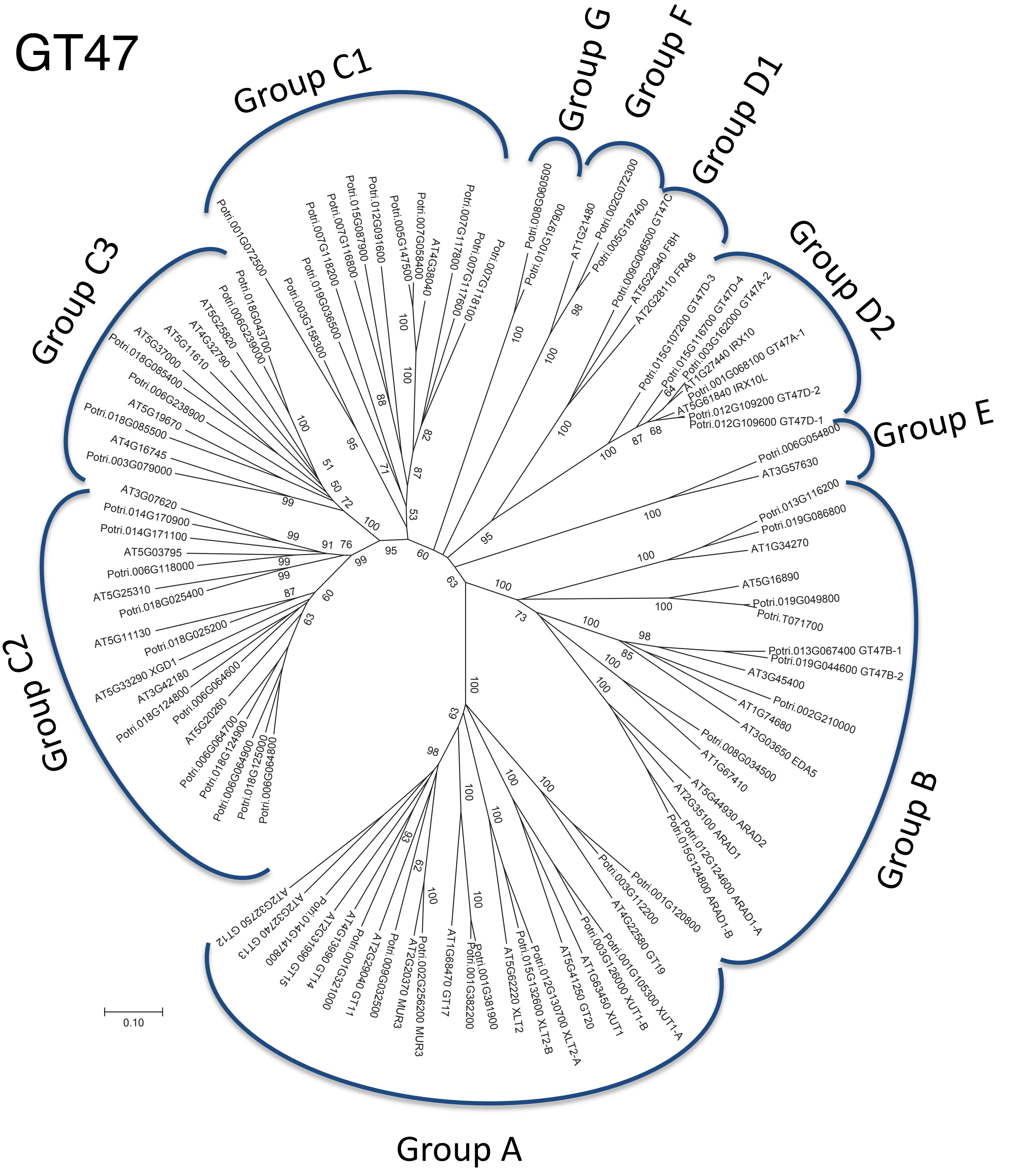
GT47
Group C1
Group G
Group F
Group D1
Group D2
Group E
Group B
Group A
Group C2
Group C3
0.10
Potri.001G072500
Potri.003G158300
Potri.019G036500
Potri.007G118200
Potri.007G116800
Potri.015G087900
Potri.012G091600
Potri.005G147500
Potri.007G058400
AT4G38040
Potri.007G117800
Potri.007G117600
Potri.007G118100
Potri.008G060500
Potri.010G197900
AT1G21480
Potri.002G072300
Potri.005G187400
Potri.009G006500 GT47C
AT5G22940 F8H
AT2G28110 FRA8
Potri.015G107200 GT47D-3
Potri.015G116700 GT47D-4
Potri.003G162000 GT47A-2
AT1G27440 IRX10
Potri.001G068100 GT47A-1
AT5G61840 IRX10L
Potri.012G109200 GT47D-2
Potri.012G109600 GT47D-1
Potri.006G054800
AT3G57630
Potri.013G116200
Potri.019G086800
AT1G34270
AT5G16890
Potri.019G049800
Potri.T071700
Potri.013G067400 GT47B-1
Potri.019G044600 GT47B-2
AT3G45400
Potri.002G210000
AT1G74680
AT3G03650 EDA5
Potri.008G034500
AT1G67410
AT5G44930 ARAD2
AT2G35100 ARAD1
Potri.012G124600 ARAD1-A
Potri.015G124800 ARAD1-B
Potri.001G120800
Potri.003G112200
AT4G22580 GT19
Potri.001G105300 XUT1-A
Potri.003G126000 XUT1-B
AT1G63450 XUT1
AT5G41250 GT20
Potri.012G130700 XLT2-A
Potri.015G132600 XLT2-B
AT5G62220 XLT2
Potri.001G381900
Potri.001G382200
AT1G68470 GT17
Potri.002G256200 MUR3
AT2G20370 MUR3
Potri.009G032500
AT2G29040 GT11
Potri.001G321000
AT4G13990 GT14
AT2G31990 GT15
Potri.014G147800
AT2G32740 GT13
AT2G32750 GT12
Potri.006G064800
Potri.018G125000
Potri.006G064900
Potri.018G124900
Potri.006G064700
AT5G20260
Potri.006G064600
Potri.018G124800
AT3G42180
AT5G33290 XGD1
Potri.018G025200
AT5G11130
Potri.018G025400
AT5G25310
Potri.006G118000
AT5G03795
Potri.014G171100
Potri.014G170900
AT3G07620
Potri.003G079000
AT4G16745
Potri.018G085500
AT5G19670
Potri.006G238900
Potri.018G085400
AT5G37000
AT5G11610
AT4G32790
AT5G25820
Potri.006G239000
Potri.018G043700

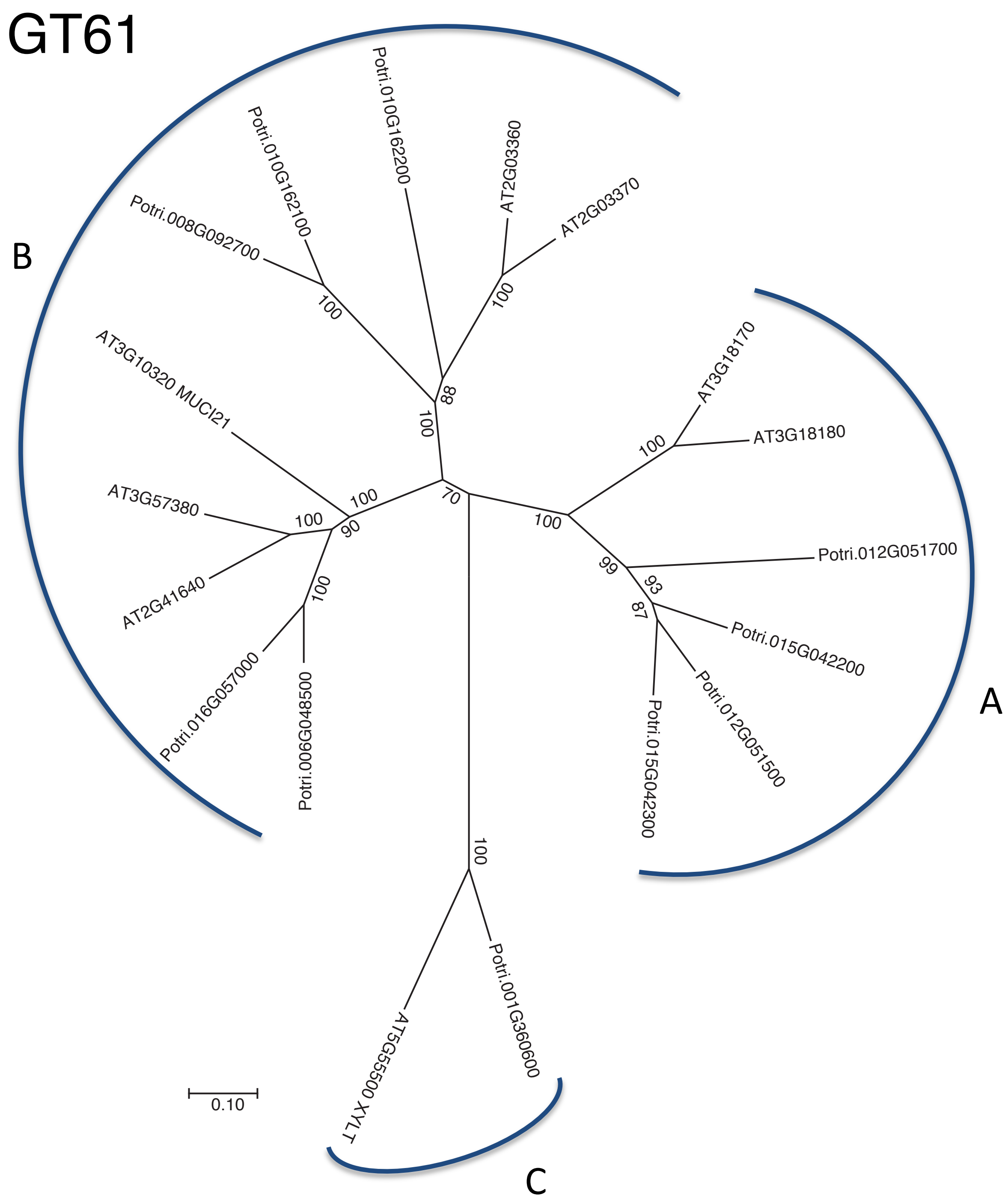
GT61
B
A
C
Potri.010G162200
AT2G03360
AT2G03370
Potri.010G162100
Potri.008G092700
AT3G10320 MUCI21
AT3G57380
AT2G41640
Potri.016G057000
Potri.006G048500
AT3G18170
AT3G18180
Potri.012G051700
Potri.015G042200
Potri.012G051500
Potri.015G042300
Potri.001G360600
AT5G55500 XYLT
0.10

GT77

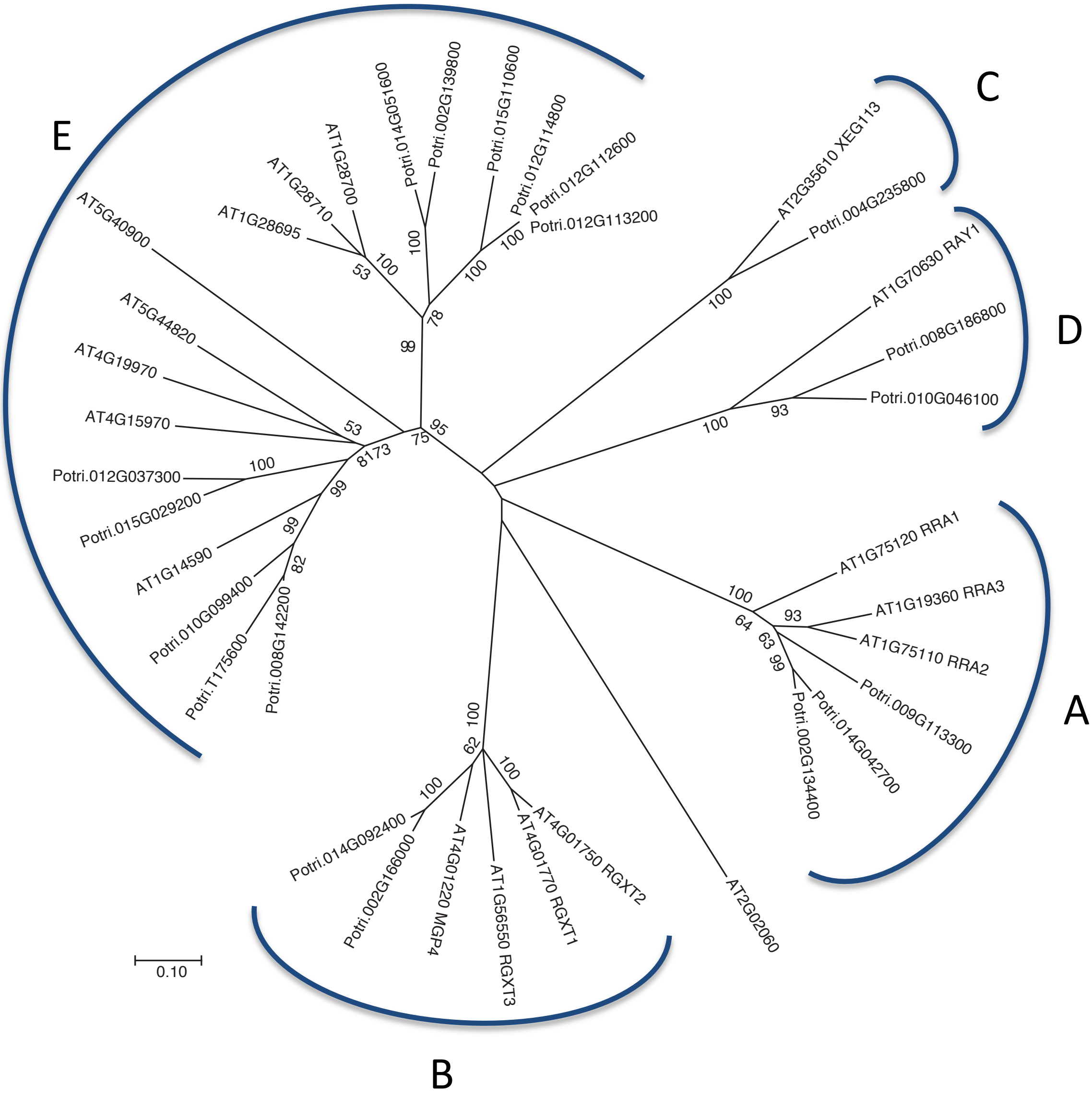
E
C
D
A
B
AT5G40900
AT1G28695
AT1G28710
AT1G28700
Potri.014G051600
Potri.002G139800
Potri.015G110600
Potri.012G114800
Potri.012G112600
Potri.012G113200
AT5G44820
AT4G19970
AT4G15970
Potri.012G037300
Potri.015G029200
AT1G14590
Potri.010G099400
Potri.T175600
Potri.008G142200
AT2G35610 XEG113
Potri.004G235800
AT1G70630 RAY1
Potri.008G186800
Potri.010G046100
AT1G75120 RRA1
AT1G19360 RRA3
AT1G75110 RRA2
Potri.009G113300
Potri.014G042700
Potri.002G134400
AT2G02060
Potri.014G092400
Potri.002G166000
AT4G01220 MGP4
AT1G56550 RGXT3
AT4G01770 RGXT1
AT4G01750 RGXT2
0.10

GT106

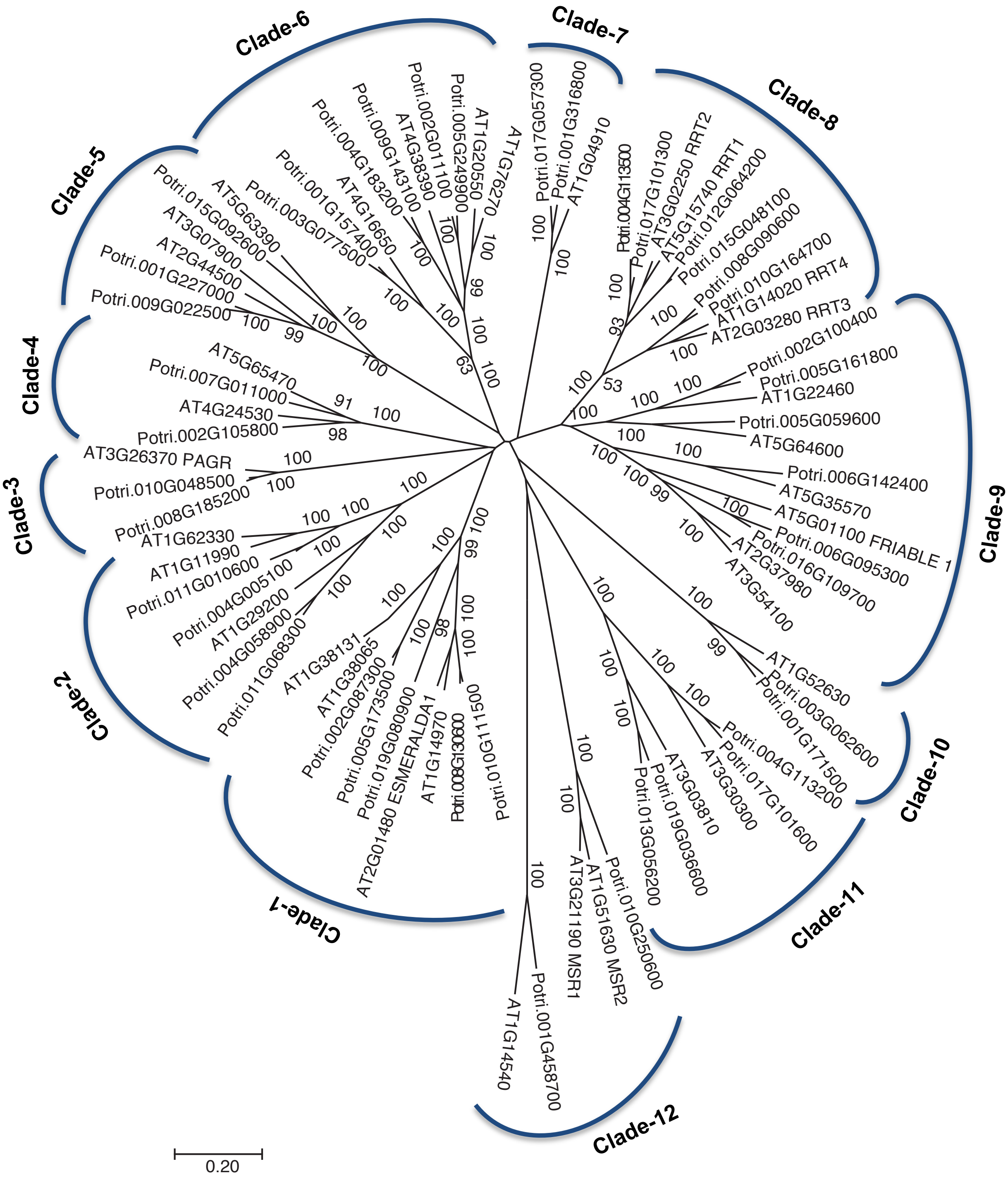

Clade-1
Clade-2
Clade-3
Clade-4
Clade-5
Clade-6
Clade-7
Clade-8
Clade-9
Clade-10
Clade-11
Clade-12
AT1G76270
AT1G20550
Potri.005G249900
Potri.002G011100
AT4G38390
Potri.009G143100
Potri.004G183200
AT4G16650
Potri.001G157400
Potri.003G077500
AT5G63390
Potri.015G092600
AT3G07900
AT2G44500
Potri.001G227000
Potri.009G022500
AT5G65470
Potri.007G011000
AT4G24530
Potri.002G105800
AT3G26370 PAGR
Potri.010G048500
Potri.008G185200
AT1G62330
AT1G11990
Potri.011G010600
Potri.004G005100
AT1G29200
Potri.004G058900
Potri.011G068300
AT1G38131
AT1G38065
Potri.002G087300
Potri.005G173500
Potri.019G080900
AT2G01480 ESMERALDA1
AT1G14970
Potri.008G130600
Potri.010G111500
AT1G14540
Potri.001G458700
AT3G21190 MSR1
AT1G51630 MSR2
Potri.010G250600
Potri.013G056200
Potri.019G036600
AT3G03810
AT3G30300
Potri.017G101600
Potri.004G113200
Potri.001G171500
Potri.003G062600
AT1G52630
AT3G54100
AT2G37980
Potri.016G109700
Potri.006G095300
AT5G01100 FRIABLE 1
AT5G35570
Potri.006G142400
AT5G64600
Potri.005G059600
AT1G22460
Potri.005G161800
Potri.002G100400
AT2G03280 RRT3
AT1G14020 RRT4
Potri.010G164700
Potri.008G090600
Potri.015G048100
Potri.012G064200
AT5G15740 RRT1
AT3G02250 RRT2
Potri.017G101300
Potri.004G113500
AT1G04910
Potri.001G316800
Potri.017G057300
0.20
