# Supplementary material for: Poplar carbohydrate‐active enzymes: whole‐genome annotation and functional analyses based on RNA expression data
Source: Plant J. 2019 Jul 1;99(4):589–609. doi: 10.1111/tpj.14417 (PMC6852159; doi:10.1111/tpj.14417)
Supplement: Supplementary file 2 — Figure S2. Variation of CAZymes expression across the wood‐forming zones of aspen. [file TPJ-99-589-s002.pdf]

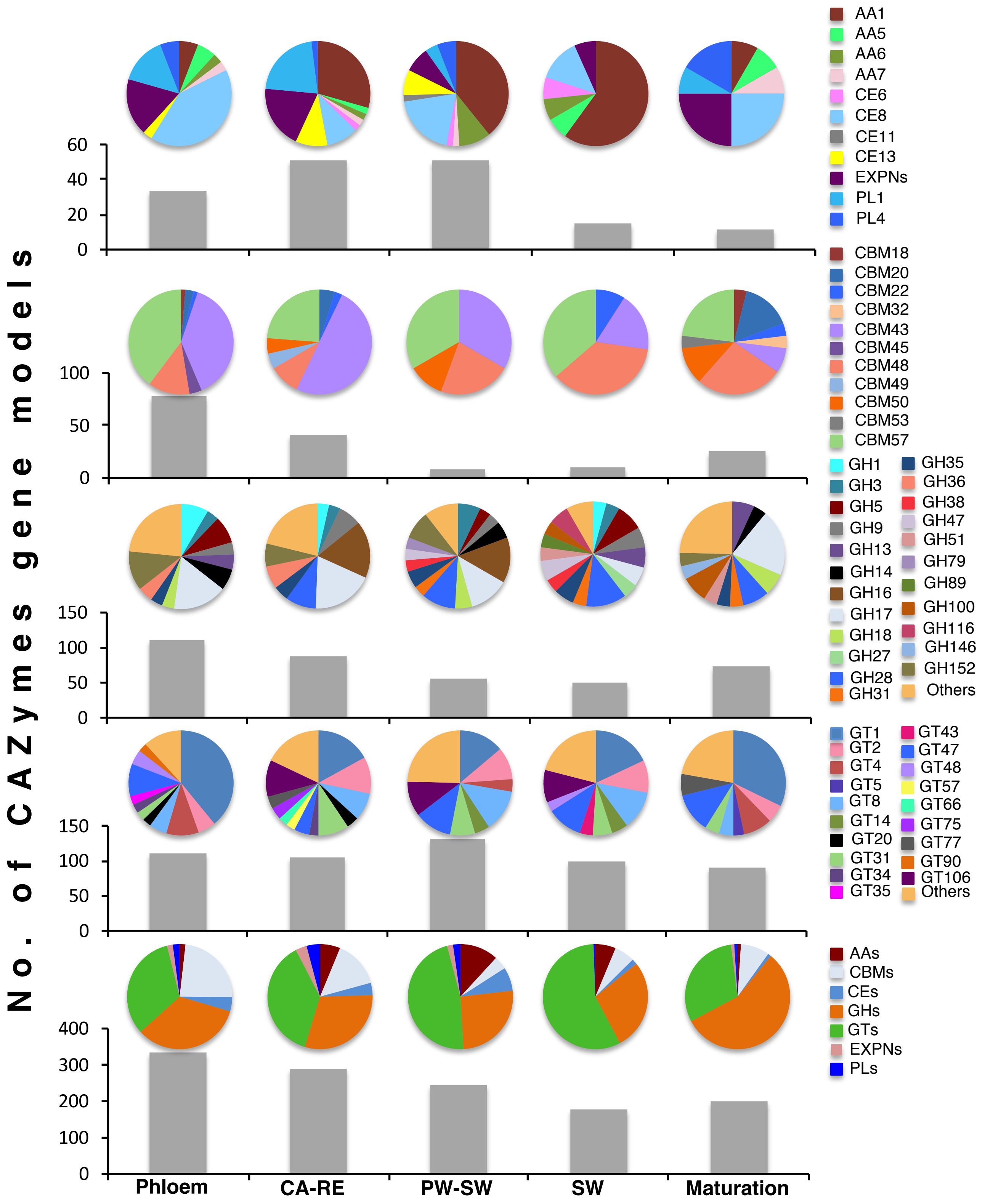

Figure S2. Variation of CAZymes expression across the wood forming zones of aspen based on AspWood (http://aspwood.popgenie.org/aspwood-v3.0/; Sundell et al. 2017). The CAZymes were grouped into expression clusters shown in Fig. 1 and corresponding to phloem, cambium-radial expansion (CA-RE), primary to secondary wall biosynthesis transition (PW-SW), secondary wall biosynthesis (SW) and maturation zone. Number of CAZyme genes assigned to each cluster is shown by bar graphs and the composition of each zone cluster is indicated in pie charts. GTs with smaller representation than 3% and GHs with smaller representation than 4% are grouped as ''Others''.
