# Supplementary material for: Poplar carbohydrate‐active enzymes: whole‐genome annotation and functional analyses based on RNA expression data
Source: Plant J. 2019 Jul 1;99(4):589–609. doi: 10.1111/tpj.14417 (PMC6852159; doi:10.1111/tpj.14417)
Supplement: Supplementary file 3 — Figure S3. Variability in cell wall composition across wood developmental zones for the second tree analyzed. [file TPJ-99-589-s003.pdf]

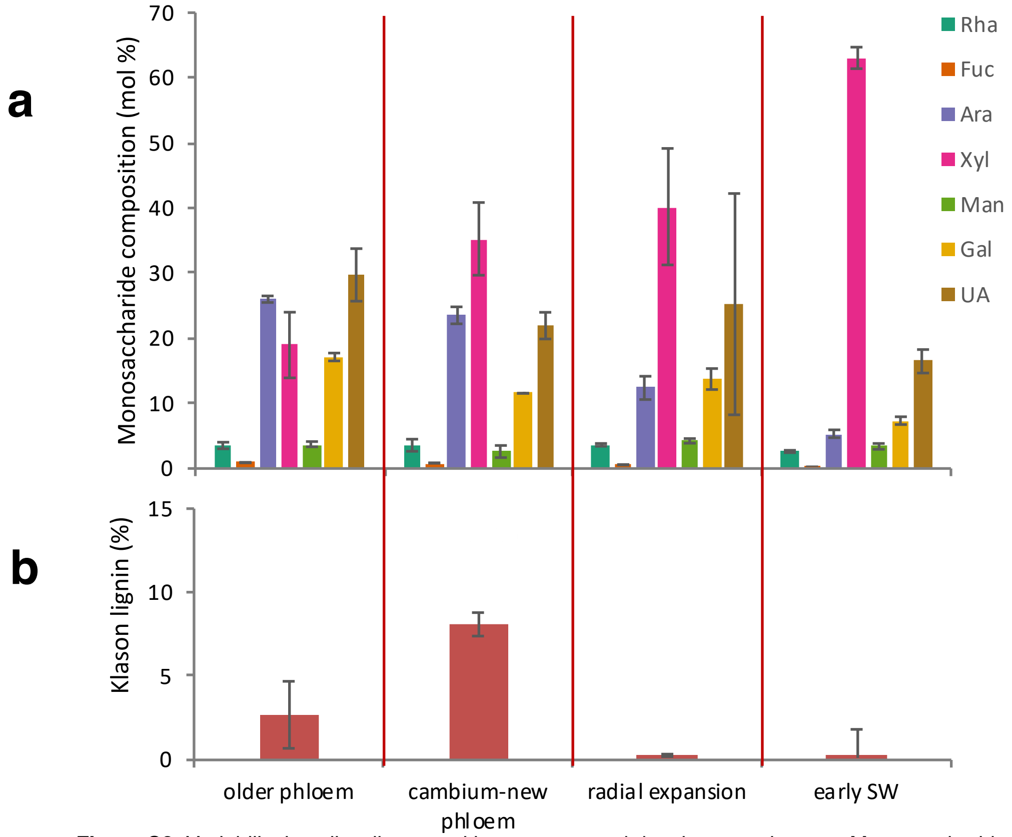

Figure S3. Variability in cell wall composition across wood developmental zones. Monosaccharide cell wall composition by alditol acetates (excluding Glc) and uronic acid contents (UA) (**a**), and Klason lignin content (**b**) in different samples of developing secondary phloem and xylem in the tree 2. Data are means, n=3 technical replicates ± SE. SW – secondary wall.
