# Supplementary material for: Poplar carbohydrate‐active enzymes: whole‐genome annotation and functional analyses based on RNA expression data
Source: Plant J. 2019 Jul 1;99(4):589–609. doi: 10.1111/tpj.14417 (PMC6852159; doi:10.1111/tpj.14417)
Supplement: Supplementary file 6 [file TPJ-99-589-s006.docx]

**LEGENDS FOR SUPPLEMENTARY TABLES AND FIGURES**

**Figure S1.** Phylogenetic trees of selected CAZyme families in poplar and *A. thaliana*. Protein sequences were aligned by MUSCLE (http://phylogeny.lirmm.fr/phylo_cgi/index.cgi) and phylogenic trees were constructed using Neighbor-Joining (NJ) method of MEGA7 in default mode with Bootstrap test of 1000 replicates. The numbers beside the branches correspond to % bootstrap values and are shown only for the main branches with at least 50 % support. The families are presented in alphabetical order.

**Figure S2.** Variation of CAZymes expression across the wood forming zones of aspen based on AspWood (http://aspwood.popgenie.org/aspwood-v3.0/; Sundell et al. 2017). The CAZymes were grouped into expression clusters shown in Fig. 1 and corresponding to phloem, cambium-radial expansion (CA-RE), primary-to-secondary wall biosynthesis transition (PW-SW), secondary wall biosynthesis (SW) and maturation zone. Number of CAZyme genes assigned to each cluster is shown by bar graphs and the composition of each zone cluster is indicated in pie charts. GTs with smaller representation than 3% and GHs with smaller representation than 5% are grouped as ‘’Others’’.

**Figure S3.** Variability in cell wall composition across wood developmental zones. Monosaccharide cell wall composition by alditol acetates (excluding Glc) and uronic acid contents (UA) (**a**), and Klason lignin content (**b**) in different samples of developing secondary phloem and xylem in tree 2. Data are means, n=3 technical replicates ± SE. SW – secondary wall.

**Table S1.** List of all annotated glycoside hydrolases (GHs) in *P. trichocarpa* genome v 3.0.

**Table S2.** List of all annotated glycosyl transferases (GTs) in *P. trichocarpa* genome v 3.0.

**Table S3.** List of all annotated polysaccharide lyases (PLs) in *P. trichocarpa* genome v 3.0.

**Table S4.** List of all annotated carbohydrate esterases (CEs) in *P. trichocarpa* genome v 3.0.

**Table S5**. List of all annotated auxiliary activities (AAs) in *P. trichocarpa* genome v 3.0.

**Table S6.** List of annotated expansins (EXPN) in *P. trichocarpa* genome v 3.0.

**Table S7.** List of all annotated carbohydrate binding motifs (CBMs) in *P. trichocarpa* genome v 3.0.

**Table S8.** *A. thaliana* auxiliary activities (AAs) listed in CAZy database and annotated based on similarity with poplar AAs. Phylogenetic analyses are shown in Fig S1.

**Table S9.** CAZyme expression matrix for comparative analyses. Variance stabilized RNA-Seq expression of all annotated CAZymes in *P. trichocarpa* genome v 3.0 in different tissues and organs. Data for developing phloem, cambium and developing xylem of hybrid aspen are based on Immanen et al. (2016), and for other tissues - on UPSC-ATLAS dataset (Sundell et al., 2015). The gene models not expressed in this combined dataset (listed at the bottom of the table) were not detected in AspWood dataset (Sundell et al., 2017) either. Dates of tissue sampling were given only for the outdoor collected material. The tissue/sample specificity score tau ranges from 0 to 1; from a ubiquitous to a specific expression, respectively. NaN= not detected; Maxn= Highest expression value (VST); NA= cannot be calculated; N-number of organs/tissues where expressed.

**Table S10.** CAZymes expressed in developing secondary xylem and phloem of aspen and their classification to expression clusters according to the AspWood (http://aspwood.popgenie.org/aspwood-v3.0/) database (Sundell et al., 2017), and to specific tissue with max. expression within the wood developmental series as shown in Figure 1. The attributes defining metabolic activity of the genes are based on Table S12.

**Table S11.** Number of CAZyme genes per family and per each expression cluster corresponding to different wood developmental zones as shown in Figure 1. Data retrieved from AspWood (http://aspwood.popgenie.org/aspwood-v3.0/; Sundell et al. 2017).

**Table S12.** List of CAZyme genes with documented and likely functions in cell wall biosynthesis and modification, and in starch and sugar metabolism.

**Table S13.** Number of CAZyme genes representing different metabolic activities per each expression cluster corresponding to different wood developmental zones as shown in Figure 1. Data retrieved from AspWood (http://aspwood.popgenie.org/aspwood-v3.0/; Sundell et al. 2017).

**Table S14.** List of genes from CAZymes-based networks in wood forming aspen tissues. The network was extracted from AspWood at threshold 5 (http://aspwood.popgenie.org/aspwood-v3.0/; Sundell et al., 2017) and the attributes defining metabolic activity of the genes are based on Table S12.

**Table S15.** List of first neighbors of primary wall associated guide genes (*PtCesA3-D, PtGAUT7-B, PtRGTX1-A, PtGT34B, PtGT43E, PtxtPL1-27* and *PtEXPA1*) from CAZymes based networks. Attributes defining metabolic activity of the genes are based on Table S12. + positive, - negative correlation.

**Table S16.** List of first neighbors of secondary wall associated guide genes (*PtCesA7-A, PtGT43B, PtGT47C* and *PtGT43C*) from CAZymes based network. Attributes defining metabolic activity of the genes are based on Table S12. + positive, - negative correlation.

**Table S17.** List of first neighbors of secondary wall expressed selected CAZymes (*PtCesA7-A,PtCesA8-A,PtGT43B,PtGT43C,PtGT47A-1,PtGATL1-B,PtGATL1-A,PtGT47C,PtCSLA1,PtGUX1-A* and *PtGUX1-B*), used as guide genes for network analysis at threshold 5 in AspWood (http://aspwood.popgenie.org/aspwood-v3.0/; Sundell et al., 2017). + positive, - negative correlation.
